# Efficient Retrieval of Images with Irregular Patterns Using Morphological Image Analysis: Applications to Industrial and Healthcare Datasets

**DOI:** 10.3390/jimaging9120277

**Published:** 2023-12-13

**Authors:** Jiajun Zhang, Georgina Cosma, Sarah Bugby, Jason Watkins

**Affiliations:** 1 Department of Computer Science, School of Science, Loughborough University, Loughborough LE11 3TT, UK; 2 Department of Physics, School of Science, Loughborough University, Loughborough LE11 3TT, UK; s.bugby@lboro.ac.uk; 3 Railston & Co., Ltd., Nottingham NG7 2TU, UK; jason@railstons.com

**Keywords:** image retrieval, morphological defect characteristics, irregular pattern analysis

## Abstract

Image retrieval is the process of searching and retrieving images from a datastore based on their visual content and features. Recently, much attention has been directed towards the retrieval of irregular patterns within industrial or healthcare images by extracting features from the images, such as deep features, colour-based features, shape-based features, and local features. This has applications across a spectrum of industries, including fault inspection, disease diagnosis, and maintenance prediction. This paper proposes an image retrieval framework to search for images containing similar irregular patterns by extracting a set of morphological features (DefChars) from images. The datasets employed in this paper contain wind turbine blade images with defects, chest computerised tomography scans with COVID-19 infections, heatsink images with defects, and lake ice images. The proposed framework was evaluated with different feature extraction methods (DefChars, resized raw image, local binary pattern, and scale-invariant feature transforms) and distance metrics to determine the most efficient parameters in terms of retrieval performance across datasets. The retrieval results show that the proposed framework using the DefChars and the Manhattan distance metric achieves a mean average precision of 80% and a low standard deviation of ±0.09 across classes of irregular patterns, outperforming alternative feature–metric combinations across all datasets. Our proposed ImR framework performed better (by 8.71%) than Super Global, a state-of-the-art deep-learning-based image retrieval approach across all datasets.

## 1. Introduction

Image Retrieval (ImR) refers to the task of searching for and retrieving relevant images from a large collection or database of images. ImR systems can also enable the search and matching of images that contain similar irregular patterns [1]. ImR has attained extensive implementation for diverse tasks, including image search engines, image captioning, and image quality assessment. An irregular pattern is a region that differs in consistency or uniformity compared to surrounding areas or a typical example baseline. Irregular pattern retrieval and analysis have been used for the detection of industrial defects [2,3,4], chest infections in medical scans [5,6,7], and ice or snow on lakes [8,9], serving industry, healthcare, and environmental monitoring. An accurate ImR system can aid experts (e.g., manufacturing engineers, doctors, and quality inspectors) during decision-making.

Many research studies have explored the retrieval of images containing irregular patterns in industrial and healthcare datasets using different features and similarity metrics. Image-based similarity metrics (e.g., Mean Squared Error (MSE), Universal Image Quality Index (UIQ) [10], Spectral Angle Mapper (SAM) [11]) [12,13,14,15], which compare the similarities between image data, provide a simple and intuitive means of comparing two images in ImR tasks. However, the similarity values computed from these metrics are sensitive to image noise and quality. Feature extraction methods can extract the hidden features of irregular patterns within images and improve retrieval performance. These methods extract Local Binary Pattern (LBP) features [16,17,18,19], Scale-Invariant Feature Transform (SIFT) features [20,21,22], as well as colour and shape features [23,24] to conduct retrieval of images with irregular patterns. Distance-based similarity metrics (e.g., Manhattan, Jaccard, Euclidean, cosine, etc.) can be utilised to compute similarity values between two sets of features extracted from images with irregular patterns.

Feature extraction is pivotal in ImR, significantly influencing the performance of a retrieval system [25,26,27]. However, many existing feature extraction methods are dataset-specific in retrieving images containing irregular patterns, resulting in low retrieval performance for different datasets [25,28]. Zhang et al. [29] proposed a set of morphological features, known as Defect Characteristics (DefChars), to characterise images with irregular patterns in terms of colour, shape, and meta aspects. Zhang et al. [29,30] successfully utilised the DefChars to reason the outputs from different Artificial Intelligence (AI)-based defect detection and classification models across industrial and healthcare datasets. This paper extends the application of the DefChars proposed by Zhang et al. [29,30] to the task of ImR. In particular, the paper proposes a novel ImR framework that extracts DefChars from images that contain irregular patterns and retrieves images with similar irregular patterns by comparing their DefChars vectors using a feature-based similarity metric.

Four datasets are employed in this study: wind turbine blade images with defects [31], chest Computerised Tomography (CT) scans with COVID-19 infections [32], heatsink images with defects [33], and lake images with ice [34]. The proposed framework was evaluated with different feature–metric combinations, such as DefChar vectors and feature-based similarity metrics, resized raw images and image-based similarity metrics (MSE, SAM, UIQ), LBP and feature-based similarity metrics, SIFT and the Euclidean metric, and a state-of-the-art Deep Learning (DL)-based ImR approach (Super Global (SG)). The retrieval results demonstrate that using the combination of DefChars and the Manhattan metric within the proposed framework consistently achieves the highest mean Average Precision (mAP) (average 0.80) and also maintains the lowest Standard Deviation (std.) (average ± 0.09) across classes of irregular patterns and a fast retrieval time (average 0.14 s per query) across all datasets. Additionally, the retrieval results also indicate that using DefChars within the proposed framework achieves relatively high and balanced retrieval accuracy across classes despite dataset imbalances or small-sized datasets. The proposed ImR framework could be expanded to various industrial tasks in the future, including irregular pattern identification, classification, and deterioration monitoring.

The contributions of this paper are as follows.

- A novel ImR framework that uses the morphological features to retrieve images containing irregular patterns.
- Retrieval and time performance comparison of the proposed ImR framework using different features (i.e., the DefChars, LBP, SIFT, and resized images) across various datasets.
- An empirical comparison of the retrieval and time performance between the proposed ImR framework and a state-of-the-art DL-based ImR approach.

There are six sections in this paper. Section 2 discusses related works concerning similarity metrics and feature extraction methods that can be employed in the ImR task. Section 3 presents the proposed ImR framework for retrieving irregular patterns in industrial and healthcare datasets. Section 4 describes the datasets, relevant feature extraction methods, similarity metrics, and the methodology used in this research, providing insight into the experimental setup. Section 5 evaluates and discusses the retrieval performance and execution time of the proposed ImR framework and compares it against a deep learning ImR framework. Section 6 summarises the key findings and conclusions drawn from the research conducted in this paper; moreover, some directions for future work are provided.

## 2. Related Works

### 2.1. Feature Extraction and Relevant Similarity Metrics for Retrieving Images

Recently, an increasing number of researchers have explored how effective feature extraction methods can enhance the performance of ImR. In 2019, Latif et al. [35] provided a comprehensive review of successful feature extraction methods used in Content-based Image Retrieval (CBIR) tasks. There are six major types of features that can be extracted from these methods, including colour-based features, shape-based features, texture-based features, spatial features, fusion features, and local features.

*Colour-based features* [36,37,38,39,40,41,42,43] offer fundamental visual information that is similar to human vision, and they are relatively robust against image transformations. *Texture-based features* [44,45,46,47,48,49,50] capture repeating patterns of local variance in image intensity; these features often hold more semantic meaning than colour-based features, though they can be susceptible to image noise. *Shape-based* features decode an object’s geometrical forms into machine-readable values and have been summarised into features that encompass contour, vertex angles, edges, polygons, spatial interrelation, moments, scale space, and shape transformation [35]. *Spatial features* [51,52,53,54,55,56,57] convey the location information of objects within the image space. *Fusion features* [58,59,60,61,62] combine basic features to form high-dimensional concatenated features—often, principal component analysis is applied to reduce dimensions. *Local features* [63,64,65,66,67,68,69,70] represent distinct structures and patches in an image, providing fine-grained details for ImR tasks.

Seetharaman and Sathiamoorthy [24] applied the Manhattan similarity metric along with colour-based and shape-based features to complete a medical ImR task. Their method achieved an average retrieval rate of 84.47% and a speed of 2.29 s. Petal et al. [23] extracted both colour-based and texture-based features from images and applied them to a CBIR task using various distance measures (e.g., Euclidean, cosine, Jaccard, Manhattan, etc.). Their approach achieved an accuracy of 87.2% in retrieving similar images. In 2022, Shamna et al. [71] employed the bag of visual words model as spatial features for retrieving medical images. Their method excelled in handling greyscale datasets, achieving a mAP of 69.70%; however, its performance on the colour datasets was lower than that of the greyscale datasets by 20.6%.

### 2.2. Recent Works to Retrieve Industrial and Healthcare Images with Irregular Patterns

In 2021, Boudani et al. [16] employed the wavelet-based LBP feature with the chi-square similarity metric to identify images containing surface defects on hot-rolled steel strips, achieving an mAP@10 score of 0.93; however, the performance was not stable between classes. Mo et al. [72] proposed a concentrated hashing method with neighbourhood embedding, utilising a Convolutional Neural Network (CNN) to extract hashing features for retrieving fabric and textile datasets in industrial applications. Their method outperformed other methods in four fabric datasets, with an average mAP of over 90%, but the precision dropped sharply, by 35%, when retrieving more than eight images. In 2022, Deep et al. [73] introduced a texture descriptor based on the concept of LBP for conducting ImR tasks on three biomedical datasets. The results showed that their proposed method reached an Average Precision (AP) rate of 91.5%; however, the AP rate of one of the datasets was very low (46%). Maintaining a consistent retrieval performance using the same feature and similarity metric for different datasets is challenging in the ImR domain. Boudani et al. [16] and Deep et al. [73] both applied LBP-based methods for ImR tasks, but their mAPs differed across their respective datasets.

Deep features, the high-level features extracted from the feature maps in DL models, have recently been used for ImR tasks. In 2022, Tan et al. [74] proposed a supervised ImR algorithm to retrieve integrated circuit images containing similar damage by matching deep features extracted from a pre-trained VGG16 model through dimension reduction and re-ranking methods. They conducted experiments to retrieve images for determining the integrated circuits’ levels (i.e., package and device) and damage classes within their levels. Their retrieval results reached a precision@5 of 97.3 when determining levels of the integrated circuits. However, the precision@5 dropped to 76.2% and 54.3% when determining the damage classes of package and device levels, respectively, resulting in an unbalanced retrieval performance of their proposed algorithms.

Agrawal et al. [5] proposed an ImR framework to retrieve chest X-ray images of lungs with COVID-19 infections. Their proposed framework extracted deep features from CNN models (i.e., VGG19 and ResNet50) and utilised distance-based metrics (i.e., chi-square, Euclidean, and cosine) to compute the similarity between images. Their retrieval performance achieved 50.4% in mAP across all classes using the ResNet50 model and cosine metric. Agrawal et al. [5] also highlighted that large-scale datasets and advanced CNN architectures can further improve retrieval performance. In 2023, Gassner et al. [75] proposed a saliency-enhanced CBIR algorithm to retrieve medical images containing skin lesions. Their proposed algorithm employed two sets of CNN classifiers to extract the deep features and saliency maps from images for the ImR tasks. The retrieval performance of their proposed algorithm improved by 0.13 points, from 0.69 to 0.82, when using saliency maps. However, the mAP of two lesion classes within the dataset was significantly lower than other classes, by 0.23 on average, due to the small number of images in these two classes.

In summary, despite the high retrieval performance achieved using deep features, some limitations still exist in conducting retrieval tasks [76]. For example, most DL-based ImR methods require training processes for extracting deep features and have expensive costs in computational resources (i.e., GPU and RAM), resulting in low efficiency when needed to update the database. In addition, there is a lack of research on DL-based ImR for industrial and healthcare datasets.

## 3. Proposed Image Retrieval Framework

**Figure 1:**
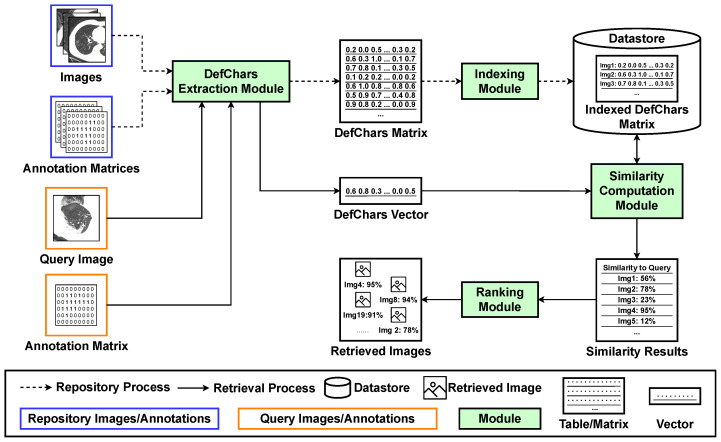
Proposed image retrieval framework.

This section introduces a proposed ImR framework, as shown in Figure 1, retrieving images with similar irregular patterns in industrial or healthcare datasets through the use of DefChars. The Python code for utilising our proposed ImR framework is provided in a GitHub repository (https://github.com/edgetrier/ImgRetreival-DefChars accessed on 12 December 2023). The proposed ImR framework consists of two main processes: the repository process and the retrieval process. The repository process employs two key modules, the DefChars extraction module and the indexing module, to construct a datastore. This datastore contains a DefChars matrix extracted from annotated images that include irregular patterns. The retrieval process, on the other hand, relies on three modules: the DefChars extraction module, the similarity computation module, and the ranking module. These modules are used to search for images with similar irregular patterns within the datastore by comparing the extracted DefChars vectors.

### 3.1. Repository Process

**DefChars Extraction module.** This module serves as a feature extraction component responsible for generating a DefChars matrix that extracts the colour-based, shape-based, and meta-based features of irregular patterns within images. The module takes as input raw images and extracts DefChars from these images. The module can also be modified to extract features using extraction methods other than DefChars, such as LBP or SIFT. The input to this module consists of a set of *images* and corresponding *annotation matrices*. Each annotation is represented as a mask-based matrix, outlining an irregular pattern’s region within an image. This matrix matches the size of the input image, with each value indicating whether the corresponding pixel in the input image falls inside or outside the irregular pattern’s region. To prepare the annotation matrices, image annotation tools like *VIA* [77], *Labelme* [78], or the *drawContours* function from the *OpenCV* package [79] can be employed if the dataset lacks annotation data. Subsequently, the module computes a DefChars vector (size: 38×1) for each image to represent the DefChars of the irregular pattern. This is achieved by analysing the pixel values within both the irregular pattern and background regions, as detailed in Section 4.2. The values in these vectors are then normalised to a range between 0 and 1. Finally, these DefChars vectors are aggregated into a *DefChars matrix*, which serves as the module’s output. The method for extracting DefChars is described in the paper by Zhang et al. [29]. Furthermore, Zhang et al. [30] also provided a toolkit for extracting the DefChars matrices from images.

**Indexing module.** This module associates each input image with its respective DefChars vector during the repository process. It takes the extracted *DefChars matrix* as input and assigns a unique index to each vector within the DefChars matrix. The index within a DefChars vector can assist users in locating the corresponding input image during the retrieval process. The module outputs an indexed DefChars matrix and stores it in a *datastore*. Additionally, this module can extend its indexing functionality when adding new images to the datastore.

### 3.2. Retrieval Process

**DefChars Extraction module.** This module is the same as the one described in Section 3.1. However, when utilised for retrieval, it extracts a single DefChars vector from an annotated query image. The input for this module consists of an *image* containing a single irregular pattern and an *annotation matrix* showing the region of the irregular pattern within the image. The annotation matrix for a query image can be collected by using an annotation tool. Moreover, preprocessing of the query image is not required in order to maintain consistency with the images in the datastore. The module generates a single *DefChars vector* that represents the features of the irregular pattern within the query image.

**Similarity computation module.** This module compares the DefChars vector extracted from the query image to each DefChars vector in the datastore using a feature-based similarity metric. In this paper, the Manhattan metric was employed as the feature-based similarity metric, although it can be substituted with any distance-based metric (e.g., cosine, Jaccard, Euclidean, etc.). The input to this module comprises the *datastore* (DefChars matrix) and the *DefChars vector* extracted from the query image. Subsequently, the module computes a set of similarity values using the selected metric to analyse the similarity between each DefChars vector in the datastore and the DefChars vector extracted from the query image. Finally, the set of similarity values is recorded and outputted in a *similarity results* table.

**Ranking module.** This module is the last step of the retrieval process, which ranks the retrieved results and presents the retrieved images. The module’s input is the *similarity results* table generated from the similarity computation module. Next, the similarity results table is ranked in order of the computed similarity values, and the module outputs the respective *images* according to their index.

## 4. Experiment Methodology

### 4.1. Datasets

Four datasets across industrial and healthcare domains were used to evaluate the retrieval performance of the proposed ImR framework: wind turbine blade defects [31], chest CT [32], heatsink defects [33] and Photi-LakeIce [34].

- The wind turbine blade dataset, provided by our industrial partner *Railston & Co., Ltd., Nottingham, United Kingdom*, contains 191 images with 304 irregular patterns across four classes (crack, void, erosion, and other). The images of wind turbine blade defects were captured during inspection; mask annotations were gathered from Zhang et al.’s experiment [31].
- The chest CT dataset was collected from Ter-Sarkisov’s [32] experiment and utilised to detect and classify the COVID-19 infection regions shown in chest CT scans. The chest CT dataset contains 750 images with 4665 irregular patterns across three classes (lung area, ground glass opacity, and consolidation), and the mask annotations were also provided in the dataset.
- The heatsink dataset was collected from Yang et al.’s experiment [33] and used to detect defects on the surfaces of gold-plated tungsten–copper alloy heatsinks. The heatsink dataset contains 1000 images, captured by an industrial camera, with 7007 irregular patterns and corresponding mask annotations across two classes (scratch and stain).
- The Photi-LakeIce (lake ice) dataset was collected from Prabha et al.’s [34] project and utilised to monitor the ice and snow on lakes by using AI techniques. The lake ice dataset contains 4017 images, captured by fixed-position webcams, with 5365 irregular patterns and corresponding mask annotations [34] across four classes (water, ice, snow, and clutter).

**Figure 2:**
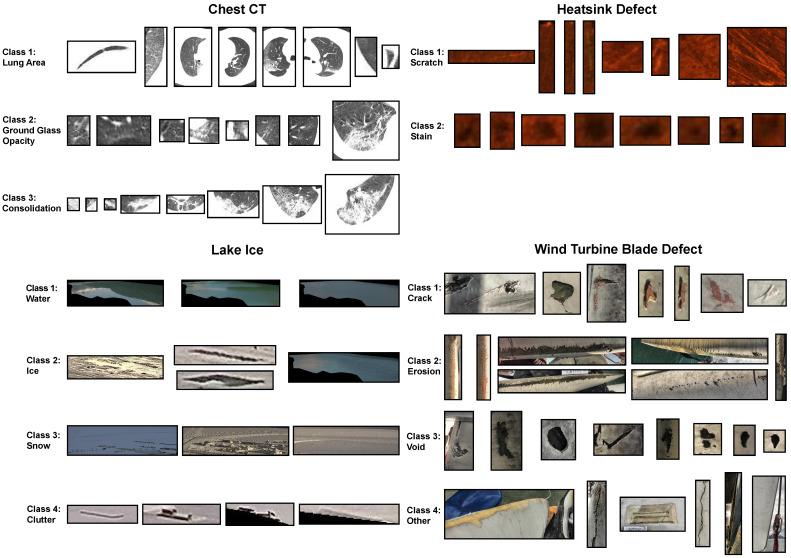
Example images of irregular patterns in each class of every dataset.

To conduct irregular pattern retrieval tasks on these datasets, each irregular pattern was cropped into an individual image based on the boundaries outlined in its respective mask annotation. The distribution of irregular pattern classes within each dataset is described in Table 1. Moreover, Figure 2 illustrates example cropped images for each class within each dataset.

### 4.2. Feature Extraction Method for Image Retrieval

**Defect characteristics:** This feature extraction methodology necessitates images with associated mask-based annotations, which are essential for calculating the DefChar values corresponding to each defect within the dataset. This is particularly important since a single image may encompass multiple defects. Table 2 provides a comprehensive list of the DefChar, their respective value ranges, and descriptions. The outcome of Zhang et al.’s method [29] manifests as a matrix with dimensions 38×n, where *n* signifies the count of defects present within the dataset.

**Scale-invariant feature transform:** Lowe [80] introduced SIFT, a local feature extraction method that can maintain an image’s scale invariance. SIFT identifies a set of keypoints and descriptors, capturing distinctive points within images. This set of keypoints and descriptors can subsequently be employed to compute similarity between images by comparing their keypoints and descriptors using the Euclidean distance metric. The SIFT extraction method contains three key steps. The first step, scale-space extrema detection, utilises the Gaussian pyramid, images are progressively downsampled to identify a collection of potential keypoints. This is achieved by analysing the differences across each level of the Gaussian pyramid. The second step, orientation assignment, involves the elimination of low-contrast keypoints, enhancing the quality of the selected keypoints. The third step, keypoint descriptor, computes dominant orientations for individual keypoints, ensuring invariance to image rotation. Lowe [80] recommended using Euclidean similarity metric (explained in Section 4.3.2) to determine the similarity between the keypoints and descriptors of two images.

**Local binary pattern:** Ojala et al. [81] introduced a texture descriptor feature extraction method designed for ImR tasks. The LBP-based method extracts texture descriptors by comparing the intensity value of each pixel in an image to the intensity values of its neighbouring pixels. The LBP extraction method contains four sequential steps. The first step, neighbourhood definition, identifies the eight neighbouring pixels around each pixel within the image. The second step, binary comparison, calculates the binary intensity value of each neighbouring pixel relative to the centre pixel. The third step, binary pattern generation, concatenates all the binary intensity values into a singular vector, following either a clockwise or counter-clockwise order. The last step, decimal representation, converts the generated binary patterns into a decimal number, serving as a representation of the texture feature.

### 4.3. Similarity Metrics for Image Retrieval

There are two categories of similarity metrics to conduct an ImR task: image-based metrics for image data and feature-based metrics for extracted feature data. Image-based metrics compute the similarity or dissimilarity between images by directly comparing the image data. The MSE, SAM, and UIQ image-based metrics are used in this experiment. Feature-based metrics assess the similarity or dissimilarity between images by analysing the extracted features. The Euclidean, cosine, Jaccard, and Manhattan feature-based metrics are used in this experiment. In the descriptions that follow, let *X* be the query image; and let *Y* be one of the retrieved images.

#### 4.3.1. Image-Based Similarity Metrics

Image similarity metrics play a crucial role in an ImR task by searching similar images within a database. Traditional image similarity metrics, such as MSE, SAM [11], UIQ [10], and Structural Similarity Index (SSIM) [13], allow for a direct comparison of pixel value differences between two images using mathematical equations.

**Mean squared error (MSE)** calculates the average squared difference in pixel values between two images. A higher MSE value signifies a greater dissimilarity between the two images.

(1) $$\mathrm{MSE}=\frac{1}{HWC}\sum_{i=1}^{H}\sum_{j=1}^{W}\sum_{k=1}^{C}{\left(x\left(i,j,k\right)-y\left(i,j,k\right)\right)}^{2}$$

where *H* represents the height of the image; *W* represents the width of the image; *C* represents the number of channels (colour components) in each pixel; x(i,j,k) represents the pixel value of the *i*th row, *j*th column, and *k*th channel in the query image *X*; y(i,j,k) represents the pixel value of the *i*th row, *j*th column, and *k*th channel in the retrieving image *Y*.

**Spectral angle mapper (SAM)** calculates the angular disparity between two spectral signatures within a high-dimensional spectral space. A higher SAM value signifies a greater dissimilarity between the two images.

(2) $$\mathrm{SAM}=\frac{1}{C}\sum_{k=1}^{C}cos^{-1}\left\{\frac{\sum_{i=1}^{H}\sum_{j=1}^{W}x\left(i,j,k\right)\;\cdot \;y\left(i,j,k\right)}{\sqrt{\sum_{i=1}^{H}\sum_{j=1}^{W}x{\left(i,j,k\right)}^{2}}\;\cdot \;\sqrt{\sum_{i=1}^{H}\sum_{j=1}^{W}y{\left(i,j,k\right)}^{2}}}\right\}$$

where *C* represents the number of channels (colour components) in each pixel; *H* represents the height of the image; *W* represents the width of the image; x(i,j,k) represents the pixel value of the *i*th row, *j*th column, and *k*th channel in the query image *X*; and y(i,j,k) represents the pixel value of the *i*th row, *j*th column, and *k*th channel in the retrieving image *Y*.

**Universal image quality index (UIQ)** takes into account the similarity between two images based on their correlation, luminance, and contrast. A higher UIQ value signifies a greater similarity between the two images. The maximum possible value of the UIQ is 1, indicating that the two images are exactly the same.

(3) $$\mathrm{UIQ}=\frac{1}{C}\sum_{k=1}^{C}\frac{\sigma_{x_{k}y_{k}}}{\sigma_{x_{k}}\sigma_{y_{k}}}\;\cdot \;\frac{2\bar{x_{k}}\bar{y_{k}}}{{\left(\bar{x_{k}}\right)}^{2}+{\left(\bar{y_{k}}\right)}^{2}}\;\cdot \;\frac{2\sigma_{x_{k}}\sigma_{y_{k}}}{\sigma_{x_{k}}^{2}+\sigma_{y_{k}}^{2}}$$

where $\bar{x_{k}}$ is the mean of the *k*th channel’s pixel values in the query image *X*; $\bar{y_{k}}$ is the mean of the *k*th channel’s pixel values in the retrieving image *Y*; $\sigma_{x_{k}}^{2}$ is the variance of the *k*th channel’s pixel values in the query image *X*; $\sigma_{y_{k}}^{2}$ is the variance of the *k*th channel’s pixel values in the retrieving image *Y*; $\sigma_{x_{k}}$ is the std. of the *k*th channel’s pixel values in the query image *X*; $\sigma_{y_{k}}$ is the std. of the *k*th channel’s pixel values in the retrieving image *Y*; $\sigma_{x_{k}y_{k}}$ is the covariance of the *k*th channel’s pixel values between the query image *X* and the retrieving image *Y*.

#### 4.3.2. Feature-Based Similarity Metrics

**Euclidean distance** calculates the direct straight-line distance between each point of two vectors. A higher value of the Euclidean distance signifies a greater dissimilarity between the two images.

(4) $$\mathrm{Euclidean}\;\mathrm{Distance}=\sqrt{\sum_{i=1}^{N}{\left(x_{i}-y_{i}\right)}^{2}}$$

where *N* represents the number of elements in the feature vector; $x_{i}$ represents the *i*th value of the extracted feature vector from the query image *X*; and $y_{i}$ represents the *i*th value of the extracted feature vector from the retrieving image *Y*.

**Cosine distance** calculates the cosine of the angles between two vectors. A higher value of the cosine distance indicates that the two images are more similar.

(5) $$\mathrm{Cosine}\;\mathrm{Distance}=\frac{\sum_{i=1}^{N}x_{i}\;\cdot \;y_{i}}{\sqrt{\sum_{i=1}^{N}x_{i}^{2}}\;\cdot \;\sqrt{\sum_{i=1}^{N}y_{i}^{2}}}$$

where *N* represents the number of elements in the feature vector; $x_{i}$ represents the *i*th value of the extracted feature vector from image *X*; and $y_{i}$ represents the *i*th value of the extracted feature vector from image *Y*.

**Manhattan distance** calculates the sum of absolute differences between corresponding elements of two vectors. A larger value of the Manhattan distance signifies that the two images are more dissimilar.

(6) $$\mathrm{Manhattan}\;\mathrm{Distance}=\sum_{i=1}^{N}\left|x_{i}-y_{i}\right|$$

where *N* represents the number of elements in the feature vector; $x_{i}$ represents the *i*th value of the extracted feature vector from the query image *X*; and $y_{i}$ represents the *i*th value of the extracted feature vector from the retrieving image *Y*.

**Jaccard distance** calculates the ratio of the common elements between two feature vectors to the total number of elements present in the vectors. A higher value of the Jaccard distance suggests that the two images are more similar in terms of the shared features or values.

(7) $$\mathrm{Jaccard}\;\mathrm{Distance}=\frac{x\cap y}{x\cup y}$$

where x∩y represents the intersection of the sets of elements present in the feature vectors of the query image *X* and retrieving image *Y*, and x∪y represents the union of the sets of elements present in the feature vectors of the query image *X* and retrieving image *Y*.

### 4.4. Methodology

The proposed ImR framework (as described in Section 3) was utilised to conduct experiments for evaluating its performance when using different feature sets and similarity metrics across four different datasets. The experiments were conducted on a high-performance computer with an AMD Ryzen 9 CPU and 32 GB RAM. The utilisation of a graphics processing unit (GPU) was not necessary for the experiments.

1. In the initial step (DefChar extraction module) of the experiment, feature sets are extracted:
  **Feature set 1** contains the raw images that have been compressed into four different sizes (i.e., 100×100, 50×50, 20×20, 8×8) with the dual goals of normalisation and acceleration of the retrieval process. Hence, feature set 1 has four subsets of features each corresponding to a different size.
  **Feature set 2** contains DefChars extracted from raw images. Raw images should be utilised when extracting DefChars, due to information loss caused by image resizing.
  **Feature sets 3 and 4** contain LBP and SIFT features extracted from each of the feature subsets described in feature set 1. The parameters of LBP were set to radius = 1, sample points = 8, and method = uniform, following the recommendations by Rahillda et al. [82] based on their experimental results. The SIFT parameters used in this experiment were set according to the guidelines by Lowe [80]: nFeatures = max, nOctaveLayers = 3, contrastThreshold = 0.3, edgeThreshold = 10, and sigma = 1.6. The feature sets are separately stored (indexing module) in a datastore.
2. In the next step (similarity computation module), the similarity between a query image and images—represented as feature vectors—found in the datastore is computed. In this experiment, each image from the datasets is iteratively selected to be a query. To compute the similarity between images, feature- and image-based similarity metrics are applied. Feature-based similarity metrics (i.e., Euclidean, cosine, Manhattan, and Jaccard) are utilised for DefChar, SIFT, and LBP features; and image-based similarity metrics (i.e., MSE, SAM, and UIQ) are utilised for compressed raw images.
3. Then, the retrieved irregular patterns are ranked (ranking module) according to the computed similarity values. The metrics described in Section 4.5 are applied to evaluate the retrieval performance.

### 4.5. Evaluation Measures

This section introduces the evaluation measures employed within the scope of an ImR task carried out in this experiment. In the context of an ImR task, the primary objective revolves around searching for relevant images with similar irregular patterns within a datastore. A relevant image to an image query is one that belongs to the same class as the query image. Precision@K is the ratio of relevant images with irregular patterns correctly retrieved among the top *K* retrieved images with irregular patterns, determining the retrieval performance for a single query. AP@K is the average value of Precision@K in all queries that are retrieved for one irregular pattern class within a dataset, determining the retrieval performance for an irregular pattern class in a dataset. Subsequently, mAP@K is the average value of AP@K across all irregular pattern classes within a dataset, determining the retrieval performance for a dataset. Furthermore, the std. values for AP and mAP were computed to evaluate the variations in the retrieval performance in classes and datasets, respectively.

## 5. Results and Discussion

This section discusses the retrieval and speed performance of the proposed ImR framework shown in Figure 1 (see Section 3) using different feature sets and similarity metrics. The experimental methodology is discussed in Section 4.4. The retrieval results when using different settings are presented in Appendix A, Appendix B, Appendix C, Appendix D. Furthermore, this section includes a comparison between the proposed ImR framework with a DL-based ImR approach.

In the rest of the paper, the following descriptions apply:

- *DefChar-based methods* refer to the proposed ImR framework using DefChars within the DefChar extraction module.
- *Image-based methods* refer to the proposed ImR framework using resized images instead of the DefChar extraction module.
- *LBP-based methods* refer to the proposed ImR framework using LBP features instead of the DefChar extraction module.
- *SIFT-based methods* refer to the proposed ImR framework using SIFT features instead of the DefChar extraction module.

### 5.1. Image Retrieval Performance When Using Different Feature Sets and Similarity Metrics across Datasets

#### 5.1.1. Chest CT Dataset

Table A1 shows the mAPs along with std. values when using different feature sets and similarity metrics in the chest CT dataset; Table A2, Table A3, Table A4 show the APs with std. values for each class within the dataset.

**ImR performance when using the proposed DefChar-based methods.** There were two similarity metrics (i.e., cosine and Euclidean) that yielded the highest mAP and the lowest std., averaging 0.85 ± 0.06. In terms of retrieval performance for each class using the cosine or Euclidean metric, both metrics had the same mAP values and std. values for class 1. For class 2, the Euclidean metric achieved a slightly higher AP of 0.01 compared to the cosine metric at @1 and @20. Conversely, the cosine metric outperformed the Euclidean metric by 0.01 in AP at @1 and @10 for class 3. Additionally, the Manhattan metric showed noteworthy retrieval performance for the chest CT dataset and relatively achieved the highest AP for classes 1 and 2. However, for class 3, the retrieval performance of the Manhattan metric was slightly lower than those of the cosine and Euclidean metrics, with an average difference of 0.01–0.02 at @5, @10, and @15. This resulted in a higher std. in mAP, despite the metrics having the same mean mAP values.

**ImR performance when using image-based methods.** The best-performing image-based methods were the UIQ metric with a 20×20 image size, averaging 0.76 ± 0.17 in terms of mAP. The UIQ metrics with 50×50 and 100×100 image sizes showed similar mAP values across the range from @1 to @20, but they had higher std. values compared to the UIQ metric with a 20×20 image size. The UIQ metric with a 20×20 image size showed a relatively small difference between the maximum and minimum AP in different classes (i.e., @1: 0.94–@20: 0.89 for class 1 and @1: 0.59–@20: 0.58 for class 3), although it may not achieve the highest AP.

**ImR performance when using LBP-based methods.** The performance of the LBP-based methods showed a correlation with the image size. The highest mAP was achieved, averaging 0.30 ± 0.22 between @1 and @20, when using images of size 100×100. However, it is worth noting that the retrieval performance of the best-performing LBP-based method for each class was not consistent.

**ImR performance when using SIFT-based methods.** The 20×20 image size proved to be the optimal setting for the SIFT-based method, with an average mAP of 0.52 ± 0.31. The std. of the SIFT-based methods were the highest among all methods; consequently, the SIFT-based methods struggled to maintain consistent retrieval performance across all classes within the chest CT dataset.

#### 5.1.2. Heatsink Dataset

Table A5 shows the mAPs, along with std., when using different feature sets and similarity metrics in the heatsink dataset; Table A6 and Table A7 show the APs with std. for each class within the dataset.

**ImR performance when using the proposed DefChar-based methods.** All similarity metrics consistently achieved the highest mAP with an average of 0.97 ± 0.02, except for the Jaccard metric. When analysing the performance for each class, the Manhattan metric sometimes reached a slightly higher std., by ±0.01, compared to the cosine and Euclidean metrics. Additionally, the AP for the Manhattan metric was sometimes lower by 0.01. As a result, the performance of the Manhattan metric was slightly lower than that of the others, particularly at @10 and @15.

**ImR performance when using image-based methods.** The average mAP between @1 and @20 reached its peak at 0.88 ± 0.08 when using 100×100 images with the UIQ metric, although its mAP@1 was slightly lower, at 0.86, compared to others. Additionally, the MSE with an 8×8 image size, demonstrated relatively high performance, with an average mAP of 0.87. When considering the performance for each class, the UIQ metric with a 100×100 image size and the MSE metric with an 8×8 image size consistently maintained a high AP across all classes. In contrast, other methods showed fluctuations in AP when applied to different classes.

**ImR performance when using LBP-based methods.** The LBP-based methods reached consistent mAP and std. values across all feature-based similarity metrics. The best-performing LBP-based methods achieved an mAP of 0.53 ± 0.18 on average when utilising 8×8 images. However, it is worth noting that the performance of the LBP-based methods varied significantly for each class. The LBP-based method with 8×8 images significantly outperformed the others, by more than 0.43 in AP for class 2; but, the performance of this metric fell behind the others for class 1, particularly when compared to the LBP-based method with a 20×20 image size.

**ImR performance when using SIFT-based methods.** The best retrieval performance was 0.54 ± 0.23 in terms of mAP when applying the 100×100 images to the SIFT-based methods. The performance of the best-performing SIFT-based methods across classes was not balanced; for instance, the AP ranged from @5: 0.51 to @20: 0.45 for class 1 and @5: 0.59 to @20: 0.64 for class 2.

#### 5.1.3. Lake Ice Dataset

Table A8 shows the mAPs, along with std., when using different feature sets and similarity metrics in the lake ice dataset; Table A9, Table A10, Table A11, Table A12 show the APs with std. for each class within the dataset.

**ImR performance when using the proposed DefChar-based methods.** The highest mAP among the DefChar-based methods had an average of 0.90 ± 0.07 when using the Manhattan metric. Also, the retrieval performance was relatively balanced across all classes within the dataset; the AP for all classes exceeded 0.94 at @1 and 0.74 at @20, which was higher than for other DefChar-based methods.

**ImR performance when using image-based methods.** All image-based methods reached similar performance; however, the SAM using 8×8 images outperformed the others with the highest mAP (0.86) and the lowest std. (±0.13). In terms of performance across different classes, there was no significant difference observed between all similarity metrics and image sizes. Nevertheless, the SAM with an 8×8 image size achieved a higher AP than other image-based methods by 0.04–0.06 for class 4, resulting in a higher mAP with a lower std. value.

**ImR performance when using LBP-based methods.** All LBP-based methods reached relatively low mAP values across all classes. The best-performing LBP-based method, when using any feature-based similarity metric with 100×100 images, only reached an average mAP of 0.26 ± 0.37. Furthermore, this best-performing LBP-based method achieved high performance for class 3 (from mAP@1: 0.99 to mAP@20: 0.68); for other classes, however, the mAP dropped significantly, falling below 0.23 and even reaching 0.00.

**ImR performance when using SIFT-based methods.** In all SIFT-based methods, using large-sized images (i.e., 100×100) achieved the highest mAP, averaging 0.64 ± 0.15. The performance of the SIFT-based method with a 100×100 image size was relatively balanced between all classes, except for class 4, where the AP was 40% lower than those for other classes.

#### 5.1.4. Wind Turbine Blade Dataset

Table A13 shows the mAPs, along with std., when using different feature sets and similarity metrics in the wind turbine blade dataset; Table A14, Table A15, Table A16, Table A17 show the APs with std. for each class within the dataset.

**ImR performance when using the proposed DefChar-based methods.** In the DefChar-based methods, the Manhattan metric outperformed others with the highest mAP (0.62) and the lowest std. (±0.17). When considering the performance for each class, the retrieval performance using the Manhattan metric generally exceeded other metrics, except for AP@15 and AP@20 in class 2, and AP@5, AP@10, and AP@15 in class 4.

**ImR performance when using image-based methods.** In the image-based methods, two settings (i.e., MSE with an 8×8 image size and UIQ with a 20×20 image size) both achieved the highest mAP (0.44) and the lowest std. (±0.31). When evaluating the performance for each class using these best-performing image-based methods, they reached similar AP values across all classes. However, the AP of the MSE metric occasionally exceeded that of the UIQ by 0.03–0.08 at @1 for all classes except class 3.

**ImR performance when using LBP-based methods.** The feature-based similarity metric with a small-sized image (i.e., 8×8) outperformed other LBP-based methods and achieved an average mAP of 0.26 ± 0.17. However, all LBP-based methods, including the best-performing one, struggled to maintain a consistent performance across all classes. For instance, the AP of the best-performing method was lower than other LBP-based methods between @1 and @20 for classes 1 and 3.

**ImR performance when using SIFT-based methods.** The highest mAP was achieved at 0.35 ± 0.30 when utilising the Euclidean metric with a 100×100 image size in the SIFT-based method. When evaluating the performance differences of the SIFT-based methods across each class, the best-performing method was relatively more accurate than others for class 3 by over 0.10, although it did not outperform the others for the rest of the classes.

### 5.2. Overall Performance Comparisons for Image Retrieval Tasks

This section compares the retrieval performance of different feature extraction methods, utilising the settings that demonstrated the best results for each dataset. These settings were selected based on the analysis presented in Section 5.1. Additionally, this section delves into investigating the time completed for each method and dataset in the ImR task.

**Figure 3:**
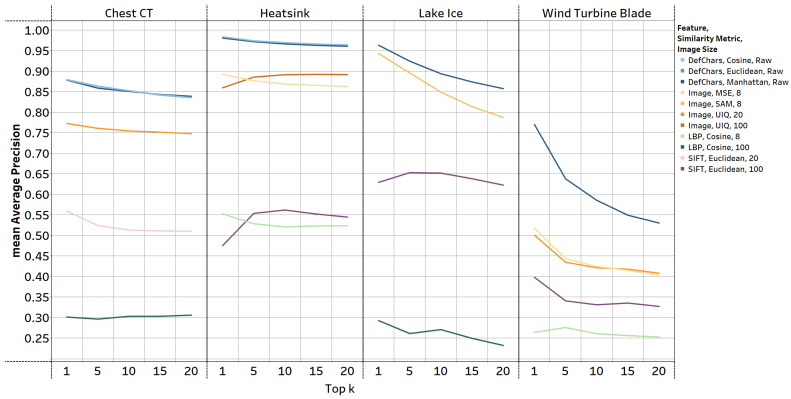
mAP of the best performing ImR methods using different features, similarity metrics, and image sizes (as shown in the legend). The blue-based, orange-based, green-based, and purple-based lines, respectively, represent the ImR methods which used the DefChars, resized image, LBP, and SIFT features. The *image size* in the legend is the size of the image that was input into the ImR approach. For example, number 8 indicates that an image of size 8×8 was used.

**Comparison of retrieval performance across datasets:** Figure 3 presents a line chart illustrating the mAP of the best-performing ImR methods, that were explained earlier, across all datasets. The DefChar-based methods consistently outperformed other methods across all datasets. When using DefChars, the performance of different similarity metrics was not significantly different; however, the Manhattan metric reached the highest mAP across all datasets. Image-based ImR methods were the second best performing across all datasets. However, the choice of similarity metrics and image sizes emerged as significant factors influencing performance for different datasets, and there is no image-based methods with a consistent setting that relatively maintains high retrieval performance. For instance, the UIQ metric performed relatively better with large-sized images in the chest CT and heatsink datasets, while the SAM and MSE metrics achieved higher mAP in the lake ice and wind turbine blade datasets when utilising small-sized images. The performance of LBP-based methods were the worst and did not show significant differences based on the choice of similarity metric. Furthermore, image sizes can easily affect the retrieval performance of LBP-based methods on different datasets. For instance, the chest CT and lake ice datasets reached higher mAP values with larger-sized images, whereas the heatsink and wind turbine blade datasets yielded better results with smaller-sized images. SIFT-based methods showed a better retrieval performance compared to the LBP-based methods. Using small-sized images (e.g., 20×20) within the SIFT-based methods reached higher retrieval performance than using large-sized images across all but the chest CT dataset.

**Figure 4:**
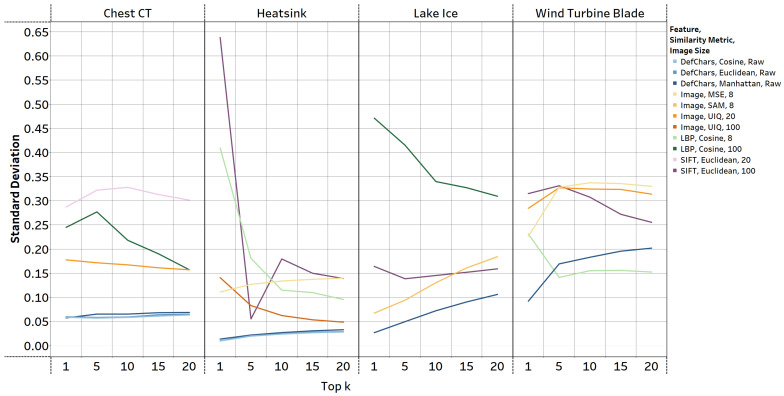
std. of the best-performing ImR methods using different features, similarity metrics, and image sizes (as shown in the legend). The blue-based, orange-based, green-based, and purple-based lines, respectively, represent the ImR methods which used DefChars, resized images, LBPs, and SIFT features. *Image size* in the legend is the size of the image that was input into the ImR approach. For example, number 8 indicates that an image of size 8×8 was used.

**Comparison of retrieval performance stability across classes and datasets:** Figure 4 provides insight into the std. values encountered when calculating the mAP using the best performing methods that were explained in Section 5.1. DefChar-based methods overall achieved the lowest std. across all datasets. This indicates their relatively stable and reliable performance. In the wind turbine blade dataset, the std. of LBP-based methods was ±0.03–0.05 lower than that of the DefChar-based methods on average. However, the mAP achieved by LBP-based methods were the lowest among all methods. In contrast, the rest of the methods, such as image-based, SIFT-based, and LBP-based methods, reached higher std. values, often exceeding those of DefChar-based methods by more than ±0.1 across datasets. Moreover, these methods had varying std. values across different datasets. This implies that these methods might show less consistency when retrieving irregular patterns from different classes within a dataset. As noted in Table 1, the datasets are relatively imbalanced between each class. However, the ImR method utilising DefChars and the Manhattan metric showed the capability to maintain relatively high and balanced accuracy in retrieving similar irregular patterns. Additionally, the performance of DefChar-based methods did not show significant deterioration when applied to a small dataset (i.e., wind turbine blades).

**Figure 5:**
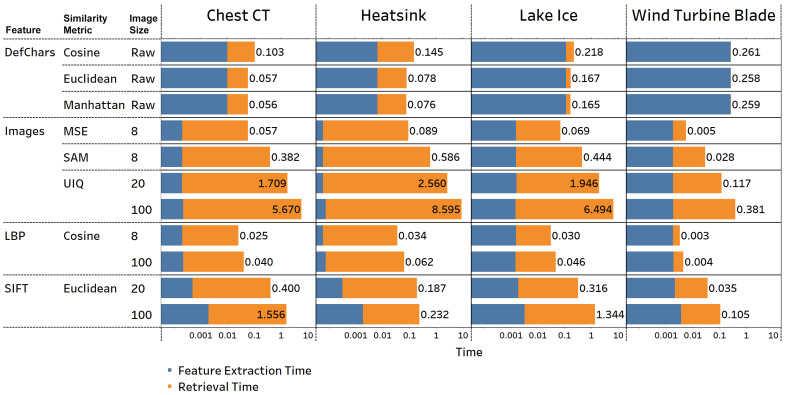
Average time (s) required to extract features (blue bars) and average retrieval time (s) (orange bars) of images across queries across four datasets. The value at the end of each bar is the total of the feature extraction and retrieval times. The *image size* column shows the size of the image that was input into the ImR approach. For example, number 8 indicates that an image of size 8×8 was used.

**Comparison of time performance across datasets:** Figure 5 presents the time required to extract features and retrieve images with similar irregular patterns for each query across four datasets. Among these methods, LBP-based methods achieved the shortest total time, taking less than 0.062 s per query. On the other hand, UIQ consumed the most time, especially when using 100×100 images, resulting in query times exceeding 5 s for large datasets such as chest CT, heatsink, and lake ice. In the DefChar-based methods, the retrieval time ranged from 0.06 to 0.26 s, making it one of the faster approaches, except for in the wind turbine blade dataset. However, while the feature extraction times for DefChar-based methods were comparatively longer than other methods, their retrieval times were significantly shorter than those of other methods. The image-based approaches generally required more time for retrieval, although image resizing reduced the feature extraction time. The SIFT-based methods needed an additional 0.0003 to 0.0018 s for extracting the SIFT features after image resizing, but the retrieval time was relatively shorter than most image-based methods. When considering the time impact of the dataset or image size, a large dataset or image size typically results in a longer execution time. For instance, the ImR time for UIQ metrics when using 100×100 images was over three times longer than when using 20×20 images. On the other hand, the heatsink dataset, the largest dataset containing 7007 irregular patterns (see Table 1), used longer retrieval times across all datasets, because the query image needed to be compared to all images in the datastore. However, DefChar-based methods outperformed all other methods in terms of retrieval performance.

### 5.3. Image Retrieval Performance Comparisons between the Proposed ImR Framework and a DL-Based ImR Framework

This section empirically evaluates the retrieval performance of the proposed ImR framework when using its optimal settings (i.e., DefChars and Manhattan metric), compared to a state-of-the-art DL-based ImR approach (i.e., SG). In 2023, Shao et al. [83] proposed a supervised DL-based ImR retrieval framework, called SG, to conduct retrieval tasks for large-scale Google landmark image datasets: the revisited Oxford5k and Paris6k [84]. Their framework trained a ResNet101 model to extract deep features from images and employed a re-ranking method to search for similar images in the database. Their proposed framework achieved a mAP of 82.9% and outperformed other state-of-the-art DL-based ImR frameworks (i.e., CVNet [85], DOLG [86] and DELG [87]) in terms of retrieval and speed performance.

Motivated by these results, this paper evaluates the retrieval and speed performance of the ImR framework proposed by Shao et al. [83] using the four datasets described in Section 4.1. The pre-trained ResNet101 model from Shao et al. [83] is directly used to extract deep features. Then, these deep features are used to retrieve images containing similar irregular patterns using their proposed re-ranking method.

The retrieval and speed performance between SG and our proposed ImR framework for each dataset are shown in Table 3 and Table 4. SG performed slightly better than ours on the wind turbine blade and lake ice datasets, by 0.01–0.07 mAP between @1 and @20. However, on the chest CT and heatsink datasets, the retrieval performance of SG was substantially lower than ours, by 0.14–0.19, in mAP. Furthermore, the std. of SG was also higher than ours across all except the lake ice dataset, demonstrating that SG was not able to achieve a high retrieval performance for all classes across datasets. Regarding speed performance, SG was significantly faster than ours, achieving 0.029–0.041 s per query across all datasets. The high retrieval speed achieved by SG can be attributed to its utilisation of a GPU to accelerate the feature extraction and retrieval processes. However, given that our proposed ImR framework runs on a CPU, the retrieval performance comparison between these two approaches in terms of speed is not directly comparable. Further benchmarking would be required with both methods running on the same hardware for an equal evaluation.

Overall, while SG has a faster retrieval speed and performs marginally better on two datasets, our proposed ImR framework achieves more robust retrieval performance across all datasets. Furthermore, our proposed ImR framework is unsupervised and does not need dataset-specific training. This highlights its capability to retrieve images with irregular patterns on different industrial and healthcare datasets without the need for customised feature learning.

## 6. Conclusions and Future Work

This paper proposed an ImR framework to retrieve images with irregular patterns and completed a comprehensive evaluation of retrieval performance using different feature extraction methods (i.e., DefChars, resized raw images, LBP, and SIFT) along with different similarity metrics across four datasets (chest CT, heatsink, lake ice, and wind turbine blade). The findings highlighted that the ImR framework, utilising DefChars and the Manhattan similarity metric, consistently demonstrated robust retrieval and speed performance across all datasets. Moreover, the proposed framework did not show a significant bias towards each class within the dataset, despite minor fluctuations in AP values possibly attributed to dataset complexity. Furthermore, the proposed ImR framework outperformed the SG, a DL-based approach, in retrieval performance across datasets.

Future work could involve comparing other DL-based ImR approaches; investigating retrieval performance when assigning weights to DefChars during the feature extraction; providing explanations for the retrieved results; and utilising object detection or segmentation techniques to automatically complete annotations of the irregular patterns for the ImR task.

## Figures and Tables

**Table 1:** Class distribution of irregular patterns in each dataset. A ‘–’ denotes that there is no such class in the dataset.

		Number of Irregular Patterns				
Dataset	Number of Images	Class 1	Class 2	Class 3	Class 4	Total Irregular Patterns
Wind Turbine Blade Defect	191	89	73	118	24	304
Lake Ice [34]	4017	606	1207	3237	315	5365
Chest CT [32]	750	2317	1668	680	–	4665
Heatsink Defect [33]	1000	2160	4927	–	–	7007

**Table 2:** Descriptions and value ranges of DefChars introduced by Zhang et al. [29].

Colour Information Extracted and Stored Separately for Irregular Patterns and Background Areas		
**DefChar Name**	**Value Range**	**Description**
Average Hue	{0,1,…,359}	Average hue value
Mode of Hue	{0,1,…,359}	Most frequent hue value
Unique Number of Hue values	{1,2,…,360}	Number of unique hue values
Hue Range	{0,1,…,180}	Difference between maximum and minimum hue value
Average Saturation	{0,1,…,254}	Average saturation value
Mode of Saturation	{0,1,…,254}	Most frequent saturation value
Unique Number of Saturation	{1,2,…,255}	Number of unique saturation values
Saturation Range	{0,1,…,254}	Difference between maximum and minimum saturation values
Average Brightness	{0,1,…,254}	Average brightness value
Mode of Brightness	{0,1,…,254}	Most frequent brightness value
Unique Number of Brightness	{1,2,…,255}	Unique brightness values
Brightness Range	{0,1,…,254}	Difference between maximum and minimum brightness value
**Colour Complexity**		
**DefChar Name**	**Value Range**	**Description**
Hue Difference	[0,1]	Hue frequency distribution difference between the defect and background areas
Saturation Difference	[0,1]	Saturation frequency distribution difference between the defect and background areas
Brightness Difference	[0,1]	Brightness frequency distribution difference between the defect and background areas
**Shape Information**		
**DefChar Name**	**Value Range**	**Description**
Number of Edges	{3,4,…}	Number of edges of the defect polygon areas
Coverage	[0,1]	Percentage of the defect polygon area covered by its bounding box
Aspect Ratio	[0,1]	Ratio between the width and height of defect bounding box
Average Turning Angles	{1,2,…,180}	Average value of vertex angles of the defect polygon area
Mode of Turning Angle	{1,2,…,180}	Value of vertex angles that appears the most often in the defect polygon
**Shape Complexity**		
Description		
Edge Ratio	[0,1]	Average length ratio between two adjacent edges in the defect polygon area
Followed Turns	[0,1]	Proportion of two adjacent vertices which turn to the same direction in the defect polygon area
Small Turns	[0,1]	Percentage of vertices which are smaller than 90${}^{\circ }$ in the defect polygon area
Reversed Turns	[0,1]	Proportion of two adjacent vertices which turn to a different direction in the defect polygon area
**Meta Information**		
**DefChar Name**	**Value Range**	**Description**
Defect Size	{1,2,…}	Number of pixels in the defect polygon area
Neighbour Distance	{0,1,2}	Categorised distances to the nearest neighbour, 0→Short (≤100 px); 1→Long; 2→No Neighbour.

**Table 3:** Retrieval performance of SG and our proposed ImR framework for each dataset. The highest mAP values with a relatively lower std. are marked in **bold text**.

Approach	mAP@1	mAP@5	mAP@10	mAP@15	mAP@20
	**Wind Turbine Blade**				
SG	**0.80 ± 0.09**	**0.66 ± 0.18**	**0.63 ± 0.19**	**0.60 ± 0.20**	**0.58 ± 0.21**
Ours	0.77 ± 0.09	0.64 ± 0.17	0.59 ± 0.18	0.55 ± 0.20	0.53 ± 0.20
	**Chest CT**				
SG	0.69 ± 0.17	0.67 ± 0.18	0.66 ± 0.18	0.65 ± 0.18	0.65 ± 0.18
Ours	**0.88 ± 0.06**	**0.86 ± 0.07**	**0.85 ± 0.07**	**0.84 ± 0.07**	**0.84 ± 0.07**
	**Heatsink**				
SG	0.84 ± 0.05	0.83 ± 0.05	0.82 ± 0.05	0.81 ± 0.05	0.81 ± 0.05
Ours	**0.98 ± 0.01**	**0.97 ± 0.02**	**0.97 ± 0.03**	**0.96 ± 0.03**	**0.96 ± 0.03**
	**Lake Ice**				
SG	**0.97 ± 0.02**	**0.96 ± 0.03**	**0.95 ± 0.04**	**0.94 ± 0.05**	**0.93 ± 0.06**
Ours	0.96 ± 0.03	0.92 ± 0.05	0.89 ± 0.07	0.87 ± 0.09	0.86 ± 0.11

**Table 4:** Speed performance (feature extraction and retrieval) of SG and our proposed ImR framework for each dataset. The second column shows the average *feature extraction time* in seconds (s) across queries. The third column shows the average *retrieval time* in seconds (s) for retrieving the results of a query; and the last column, *total time*, is the sum of columns 2 and 3.

Approach	Feature Extraction Time (s)	Average Retrieval Time (s)	Total Time (s)
	**Wind Turbine Blade**		
SG	0.039	0.0000032	0.039
Ours	0.255	0.004	0.259
	**Chest CT**		
SG	0.029	0.00000019	0.029
Ours	0.010	0.046	0.056
	**Heatsink**		
SG	0.029	0.00000019	0.029
Ours	0.006	0.070	0.076
	**Lake Ice**		
SG	0.041	0.00000019	0.041
Ours	0.112	0.053	0.165

## Data Availability

Chest CT COVID-19 Dataset: Publicly available datasets were analysed in this study. These data can be found here: http://ncov-ai.big.ac.cn/download, accessed on 30 September 2023. Heatsink Defect Dataset: Publicly available datasets were analysed in this study. These data can be found here: https://www.kaggle.com/datasets/kaifengyang/heat-sink-surface-defect-dataset, accessed on 30 September 2023. Lake Ice Dataset: Publicly available datasets were analysed in this study. These data can be found here: https://github.com/prs-eth/photi-lakeice-dataset/, accessed on 30 September 2023. Wind Turbine Blade Defect Dataset: Not applicable.

## Abbreviations

The following abbreviations are used in this manuscript:

ImR	Image Retrieval
AI	Artificial Intelligence
CBIR	Content-based Image Retrieval
CNN	Convolutional Neural Network
DL	Deep Learning
mAP	Mean Average Precision
AP	Average Precision
LBP	Local Binary Pattern
DefChars	Defect Characteristics
SIFT	Scale-Invariant Feature Transform
MSE	Mean Squared Error
SAM	Spectral Angle Mapper
UIQ	Universal Image Quality Index
SSIM	Structural Similarity Index
CT	Computerised Tomography
SG	Super Global
Std	Standard Deviation

## Appendix A. ImR Evaluation Results for the Chest CT Dataset

**Table A1:** mAP and std. values. The highest mAP with a relatively lower std. is marked in **bold text**.

Feature	Similarity Metric	Image Size	mAP@1	mAP@5	mAP@10	mAP@15	mAP@20	Average
DefChars	Cosine	Raw	**0.88 ± 0.06**	**0.86 ± 0.06**	**0.85 ± 0.06**	**0.84 ± 0.06**	**0.84 ± 0.06**	**0.85 ± 0.06**
DefChars	Euclidean	Raw	**0.88 ± 0.06**	**0.86 ± 0.06**	**0.85 ± 0.06**	**0.84 ± 0.06**	**0.84 ± 0.06**	**0.85 ± 0.06**
DefChars	Jaccard	Raw	0.51 ± 0.19	0.49 ± 0.20	0.47 ± 0.21	0.47 ± 0.21	0.47 ± 0.22	0.48 ± 0.21
DefChars	Manhattan	Raw	**0.88 ± 0.06**	0.86 ± 0.07	0.85 ± 0.07	0.84 ± 0.07	0.84 ± 0.07	0.85 ± 0.07
Image	MSE	8	**0.76 ± 0.22**	**0.74 ± 0.22**	**0.73 ± 0.22**	**0.72 ± 0.22**	**0.71 ± 0.22**	**0.73 ± 0.22**
Image	MSE	20	0.75 ± 0.24	0.74 ± 0.23	0.73 ± 0.23	0.72 ± 0.23	0.71 ± 0.23	0.73 ± 0.23
Image	MSE	50	0.75 ± 0.23	0.73 ± 0.23	0.73 ± 0.23	0.72 ± 0.23	0.71 ± 0.23	0.73 ± 0.23
Image	MSE	100	0.75 ± 0.23	0.73 ± 0.23	0.72 ± 0.23	0.72 ± 0.23	0.71 ± 0.23	0.73 ± 0.23
Image	SAM	8	**0.72 ± 0.20**	**0.69 ± 0.22**	**0.67 ± 0.22**	**0.66 ± 0.23**	**0.65 ± 0.23**	**0.68 ± 0.22**
Image	SAM	20	0.72 ± 0.21	**0.69 ± 0.22**	0.67 ± 0.23	0.66 ± 0.24	0.65 ± 0.24	0.68 ± 0.23
Image	SAM	50	0.72 ± 0.21	0.69 ± 0.23	0.67 ± 0.24	0.66 ± 0.24	0.65 ± 0.25	0.68 ± 0.23
Image	SAM	100	0.72 ± 0.21	0.69 ± 0.22	0.67 ± 0.24	0.66 ± 0.24	0.65 ± 0.25	0.68 ± 0.23
Image	UIQ	8	0.33 ± 0.58	0.33 ± 0.42	0.33 ± 0.42	0.33 ± 0.31	0.33 ± 0.23	0.33 ± 0.39
Image	UIQ	20	**0.77 ± 0.18**	**0.76 ± 0.17**	**0.75 ± 0.17**	**0.75 ± 0.16**	**0.75 ± 0.16**	**0.76 ± 0.17**
Image	UIQ	50	0.77 ± 0.20	0.76 ± 0.19	0.75 ± 0.19	0.75 ± 0.19	0.74 ± 0.19	0.75 ± 0.19
Image	UIQ	100	0.77 ± 0.21	0.76 ± 0.20	0.75 ± 0.21	0.74 ± 0.21	0.74 ± 0.21	0.75 ± 0.21
LBP	Cosine	8	0.22 ± 0.16	0.22 ± 0.17	0.22 ± 0.18	0.22 ± 0.18	0.23 ± 0.19	0.22 ± 0.18
LBP	Cosine	20	0.23 ± 0.28	0.21 ± 0.27	0.21 ± 0.28	0.21 ± 0.28	0.21 ± 0.28	0.21 ± 0.28
LBP	Cosine	50	0.28 ± 0.28	0.29 ± 0.11	0.28 ± 0.12	0.28 ± 0.08	0.28 ± 0.08	0.28 ± 0.13
LBP	Cosine	100	**0.30 ± 0.24**	**0.30 ± 0.28**	**0.30 ± 0.22**	**0.30 ± 0.19**	**0.31 ± 0.16**	**0.30 ± 0.22**
LBP	Euclidean	8	0.22 ± 0.16	0.22 ± 0.17	0.22 ± 0.18	0.22 ± 0.18	0.23 ± 0.19	0.22 ± 0.18
LBP	Euclidean	20	0.23 ± 0.28	0.21 ± 0.27	0.21 ± 0.28	0.21 ± 0.28	0.21 ± 0.28	0.21 ± 0.28
LBP	Euclidean	50	0.28 ± 0.28	0.29 ± 0.11	0.28 ± 0.12	0.28 ± 0.08	0.28 ± 0.08	0.28 ± 0.13
LBP	Euclidean	100	**0.30 ± 0.24**	**0.30 ± 0.28**	**0.30 ± 0.22**	**0.30 ± 0.19**	**0.31 ± 0.16**	**0.30 ± 0.22**
LBP	Jaccard	8	0.22 ± 0.16	0.22 ± 0.17	0.22 ± 0.18	0.22 ± 0.18	0.23 ± 0.19	0.22 ± 0.18
LBP	Jaccard	20	0.23 ± 0.28	0.21 ± 0.27	0.21 ± 0.28	0.21 ± 0.28	0.21 ± 0.28	0.21 ± 0.28
LBP	Jaccard	50	0.28 ± 0.28	0.29 ± 0.11	0.28 ± 0.12	0.28 ± 0.08	0.28 ± 0.08	0.28 ± 0.13
LBP	Jaccard	100	**0.30 ± 0.24**	**0.30 ± 0.28**	**0.30 ± 0.22**	**0.30 ± 0.19**	**0.31 ± 0.16**	**0.30 ± 0.22**
LBP	Manhattan	8	0.22 ± 0.16	0.22 ± 0.17	0.22 ± 0.18	0.22 ± 0.18	0.23 ± 0.19	0.22 ± 0.18
LBP	Manhattan	20	0.23 ± 0.28	0.21 ± 0.27	0.21 ± 0.28	0.21 ± 0.28	0.21 ± 0.28	0.21 ± 0.28
LBP	Manhattan	50	0.28 ± 0.28	0.29 ± 0.11	0.28 ± 0.12	0.28 ± 0.08	0.28 ± 0.08	0.28 ± 0.13
LBP	Manhattan	100	**0.30 ± 0.24**	**0.30 ± 0.28**	**0.30 ± 0.22**	**0.30 ± 0.19**	**0.31 ± 0.16**	**0.30 ± 0.22**
SIFT	Euclidean	8	0.40 ± 0.51	0.35 ± 0.41	0.35 ± 0.42	0.36 ± 0.33	0.36 ± 0.26	0.36 ± 0.39
SIFT	Euclidean	20	**0.56 ± 0.29**	**0.52 ± 0.32**	**0.51 ± 0.33**	**0.51 ± 0.31**	**0.51 ± 0.30**	**0.52 ± 0.31**
SIFT	Euclidean	50	0.43 ± 0.40	0.44 ± 0.39	0.45 ± 0.39	0.45 ± 0.39	0.44 ± 0.38	0.44 ± 0.39
SIFT	Euclidean	100	0.38 ± 0.39	0.40 ± 0.35	0.42 ± 0.34	0.43 ± 0.34	0.43 ± 0.33	0.41 ± 0.35

**Table A2:** AP and std. values for class 1. The highest AP with a relatively lower std. is marked in **bold text**.

Feature	Similarity Metric	Image Size	AP@1	AP@5	AP@10	AP@15	AP@20
DefChars	Cosine	Raw	0.93 ± 0.25	**0.92 ± 0.21**	0.91 ± 0.21	0.90 ± 0.21	0.90 ± 0.21
DefChars	Euclidean	Raw	0.93 ± 0.25	**0.92 ± 0.21**	0.91 ± 0.21	0.90 ± 0.21	0.90 ± 0.21
DefChars	Jaccard	Raw	0.70 ± 0.46	0.69 ± 0.33	0.68 ± 0.30	0.68 ± 0.29	0.68 ± 0.28
DefChars	Manhattan	Raw	**0.94 ± 0.24**	**0.92 ± 0.21**	**0.92 ± 0.20**	**0.91 ± 0.20**	**0.91 ± 0.20**
Image	MSE	8	0.93 ± 0.25	0.92 ± 0.20	0.91 ± 0.20	0.90 ± 0.20	0.89 ± 0.20
Image	MSE	20	0.94 ± 0.24	0.93 ± 0.19	0.92 ± 0.19	0.91 ± 0.19	**0.91 ± 0.19**
Image	MSE	50	**0.94 ± 0.23**	**0.93 ± 0.18**	**0.92 ± 0.18**	**0.92 ± 0.19**	**0.91 ± 0.19**
Image	MSE	100	0.94 ± 0.24	**0.93 ± 0.18**	**0.92 ± 0.18**	**0.92 ± 0.19**	**0.91 ± 0.19**
Image	SAM	8	0.94 ± 0.23	0.94 ± 0.17	0.93 ± 0.16	0.92 ± 0.16	0.92 ± 0.16
Image	SAM	20	**0.96 ± 0.20**	**0.95 ± 0.15**	**0.94 ± 0.15**	0.93 ± 0.15	**0.93 ± 0.15**
Image	SAM	50	**0.96 ± 0.20**	**0.95 ± 0.15**	**0.94 ± 0.15**	**0.94 ± 0.14**	**0.93 ± 0.15**
Image	SAM	100	**0.96 ± 0.20**	**0.95 ± 0.15**	**0.94 ± 0.15**	0.94 ± 0.15	**0.93 ± 0.15**
Image	UIQ	8	0.00 ± 0.00	0.80 ± 0.00	0.80 ± 0.00	0.67 ± 0.00	0.55 ± 0.00
Image	UIQ	20	0.94 ± 0.23	0.92 ± 0.19	0.91 ± 0.19	0.90 ± 0.19	0.89 ± 0.20
Image	UIQ	50	0.95 ± 0.23	0.93 ± 0.18	0.92 ± 0.18	0.91 ± 0.18	0.91 ± 0.19
Image	UIQ	100	**0.95 ± 0.22**	**0.94 ± 0.17**	**0.93 ± 0.17**	**0.92 ± 0.18**	**0.91 ± 0.18**
LBP	Cosine	8	0.40 ± 0.49	0.41 ± 0.39	0.43 ± 0.36	0.43 ± 0.35	0.43 ± 0.34
LBP	Cosine	20	**0.55 ± 0.50**	**0.53 ± 0.38**	**0.53 ± 0.36**	**0.53 ± 0.35**	**0.54 ± 0.34**
LBP	Cosine	50	0.04 ± 0.20	0.28 ± 0.20	0.27 ± 0.20	0.26 ± 0.20	0.27 ± 0.20
LBP	Cosine	100	0.03 ± 0.16	0.16 ± 0.17	0.23 ± 0.12	0.26 ± 0.12	0.27 ± 0.12
LBP	Euclidean	8	0.40 ± 0.49	0.41 ± 0.39	0.43 ± 0.36	0.43 ± 0.35	0.43 ± 0.34
LBP	Euclidean	20	**0.55 ± 0.50**	**0.53 ± 0.38**	**0.53 ± 0.36**	**0.53 ± 0.35**	**0.54 ± 0.34**
LBP	Euclidean	50	0.04 ± 0.20	0.28 ± 0.20	0.27 ± 0.20	0.26 ± 0.20	0.27 ± 0.20
LBP	Euclidean	100	0.03 ± 0.16	0.16 ± 0.17	0.23 ± 0.12	0.26 ± 0.12	0.27 ± 0.12
LBP	Jaccard	8	0.40 ± 0.49	0.41 ± 0.39	0.43 ± 0.36	0.43 ± 0.35	0.43 ± 0.34
LBP	Jaccard	20	**0.55 ± 0.50**	**0.53 ± 0.38**	**0.53 ± 0.36**	**0.53 ± 0.35**	**0.54 ± 0.34**
LBP	Jaccard	50	0.04 ± 0.20	0.28 ± 0.20	0.27 ± 0.20	0.26 ± 0.20	0.27 ± 0.20
LBP	Jaccard	100	0.03 ± 0.16	0.16 ± 0.17	0.23 ± 0.12	0.26 ± 0.12	0.27 ± 0.12
LBP	Manhattan	8	0.40 ± 0.49	0.41 ± 0.39	0.43 ± 0.36	0.43 ± 0.35	0.43 ± 0.34
LBP	Manhattan	20	**0.55 ± 0.50**	**0.53 ± 0.38**	**0.53 ± 0.36**	**0.53 ± 0.35**	**0.54 ± 0.34**
LBP	Manhattan	50	0.04 ± 0.20	0.28 ± 0.20	0.27 ± 0.20	0.26 ± 0.20	0.27 ± 0.20
LBP	Manhattan	100	0.03 ± 0.16	0.16 ± 0.17	0.23 ± 0.12	0.26 ± 0.12	0.27 ± 0.12
SIFT	Euclidean	8	0.19 ± 0.40	0.82 ± 0.10	0.82 ± 0.09	0.72 ± 0.12	0.63 ± 0.16
SIFT	Euclidean	20	0.77 ± 0.42	0.86 ± 0.20	0.86 ± 0.18	0.84 ± 0.18	0.82 ± 0.19
SIFT	Euclidean	50	**0.88 ± 0.32**	**0.89 ± 0.17**	**0.89 ± 0.15**	**0.88 ± 0.15**	**0.88 ± 0.15**
SIFT	Euclidean	100	0.83 ± 0.38	0.81 ± 0.21	0.80 ± 0.17	0.81 ± 0.16	0.81 ± 0.16

**Table A3:** AP and std. values for class 2. The highest AP with a relatively lower std. is marked in **bold text**.

Feature	Similarity Metric	Image Size	AP@1	AP@5	AP@10	AP@15	AP@20
DefChars	Cosine	Raw	0.88 ± 0.32	0.86 ± 0.24	0.85 ± 0.23	0.84 ± 0.23	0.83 ± 0.23
DefChars	Euclidean	Raw	**0.89 ± 0.31**	0.86 ± 0.24	**0.85 ± 0.22**	0.84 ± 0.22	0.84 ± 0.22
DefChars	Jaccard	Raw	0.50 ± 0.50	0.48 ± 0.25	0.47 ± 0.19	0.47 ± 0.17	0.47 ± 0.15
DefChars	Manhattan	Raw	0.88 ± 0.33	**0.86 ± 0.23**	**0.85 ± 0.22**	**0.85 ± 0.22**	**0.84 ± 0.21**
Image	MSE	8	**0.82 ± 0.38**	**0.80 ± 0.28**	**0.79 ± 0.26**	**0.78 ± 0.25**	**0.78 ± 0.25**
Image	MSE	20	0.82 ± 0.39	0.79 ± 0.28	0.78 ± 0.26	**0.78 ± 0.25**	0.77 ± 0.25
Image	MSE	50	0.81 ± 0.39	0.79 ± 0.29	0.78 ± 0.26	**0.78 ± 0.25**	0.77 ± 0.25
Image	MSE	100	0.81 ± 0.39	0.79 ± 0.29	0.78 ± 0.26	**0.78 ± 0.25**	0.77 ± 0.25
Image	SAM	8	**0.66 ± 0.47**	**0.61 ± 0.34**	**0.57 ± 0.32**	**0.55 ± 0.31**	**0.53 ± 0.30**
Image	SAM	20	0.63 ± 0.48	0.58 ± 0.36	0.55 ± 0.33	0.52 ± 0.32	0.51 ± 0.31
Image	SAM	50	0.63 ± 0.48	0.58 ± 0.36	0.54 ± 0.33	0.52 ± 0.32	0.50 ± 0.31
Image	SAM	100	0.63 ± 0.48	0.58 ± 0.36	0.54 ± 0.33	0.52 ± 0.32	0.50 ± 0.31
Image	UIQ	8	**1.00 ± 0.02**	0.20 ± 0.00	0.20 ± 0.00	0.27 ± 0.00	0.35 ± 0.00
Image	UIQ	20	0.79 ± 0.41	0.78 ± 0.28	0.77 ± 0.26	0.77 ± 0.25	0.77 ± 0.24
Image	UIQ	50	0.82 ± 0.39	**0.80 ± 0.27**	**0.79 ± 0.26**	**0.79 ± 0.25**	**0.78 ± 0.25**
Image	UIQ	100	0.82 ± 0.39	**0.80 ± 0.27**	**0.79 ± 0.26**	**0.79 ± 0.25**	**0.78 ± 0.25**
LBP	Cosine	8	0.14 ± 0.35	0.15 ± 0.23	0.16 ± 0.21	0.17 ± 0.21	0.17 ± 0.21
LBP	Cosine	20	0.09 ± 0.28	0.06 ± 0.17	0.07 ± 0.15	0.07 ± 0.15	0.07 ± 0.14
LBP	Cosine	50	0.21 ± 0.41	0.41 ± 0.24	0.40 ± 0.23	0.36 ± 0.23	0.36 ± 0.24
LBP	Cosine	100	**0.38 ± 0.49**	**0.62 ± 0.29**	**0.55 ± 0.26**	**0.51 ± 0.23**	**0.48 ± 0.23**
LBP	Euclidean	8	0.14 ± 0.35	0.15 ± 0.23	0.16 ± 0.21	0.17 ± 0.21	0.17 ± 0.21
LBP	Euclidean	20	0.09 ± 0.28	0.06 ± 0.17	0.07 ± 0.15	0.07 ± 0.15	0.07 ± 0.14
LBP	Euclidean	50	0.21 ± 0.41	0.41 ± 0.24	0.40 ± 0.23	0.36 ± 0.23	0.36 ± 0.24
LBP	Euclidean	100	**0.38 ± 0.49**	**0.62 ± 0.29**	**0.55 ± 0.26**	**0.51 ± 0.23**	**0.48 ± 0.23**
LBP	Jaccard	8	0.14 ± 0.35	0.15 ± 0.23	0.16 ± 0.21	0.17 ± 0.21	0.17 ± 0.21
LBP	Jaccard	20	0.09 ± 0.28	0.06 ± 0.17	0.07 ± 0.15	0.07 ± 0.15	0.07 ± 0.14
LBP	Jaccard	50	0.21 ± 0.41	0.41 ± 0.24	0.40 ± 0.23	0.36 ± 0.23	0.36 ± 0.24
LBP	Jaccard	100	**0.38 ± 0.49**	**0.62 ± 0.29**	**0.55 ± 0.26**	**0.51 ± 0.23**	**0.48 ± 0.23**
LBP	Manhattan	8	0.14 ± 0.35	0.15 ± 0.23	0.16 ± 0.21	0.17 ± 0.21	0.17 ± 0.21
LBP	Manhattan	20	0.09 ± 0.28	0.06 ± 0.17	0.07 ± 0.15	0.07 ± 0.15	0.07 ± 0.14
LBP	Manhattan	50	0.21 ± 0.41	0.41 ± 0.24	0.40 ± 0.23	0.36 ± 0.23	0.36 ± 0.24
LBP	Manhattan	100	**0.38 ± 0.49**	**0.62 ± 0.29**	**0.55 ± 0.26**	**0.51 ± 0.23**	**0.48 ± 0.23**
SIFT	Euclidean	8	**0.98 ± 0.15**	0.20 ± 0.05	0.20 ± 0.03	0.27 ± 0.02	0.35 ± 0.02
SIFT	Euclidean	20	0.67 ± 0.47	**0.50 ± 0.30**	**0.48 ± 0.26**	**0.49 ± 0.24**	**0.50 ± 0.21**
SIFT	Euclidean	50	0.25 ± 0.43	0.28 ± 0.24	0.30 ± 0.20	0.31 ± 0.18	0.31 ± 0.18
SIFT	Euclidean	100	0.18 ± 0.38	0.25 ± 0.23	0.28 ± 0.20	0.30 ± 0.18	0.31 ± 0.17

**Table A4:** AP and std. values for class 3. The highest AP with a relatively lower std. is marked in **bold text**.

Feature	Similarity Metric	Image Size	AP@1	AP@5	AP@10	AP@15	AP@20
DefChars	Cosine	Raw	0.82 ± 0.39	**0.81 ± 0.26**	**0.80 ± 0.24**	**0.78 ± 0.23**	**0.77 ± 0.22**
DefChars	Euclidean	Raw	0.81 ± 0.39	0.81 ± 0.27	0.79 ± 0.24	**0.78 ± 0.23**	0.77 ± 0.23
DefChars	Jaccard	Raw	0.33 ± 0.47	0.29 ± 0.21	0.26 ± 0.15	0.25 ± 0.12	0.25 ± 0.11
DefChars	Manhattan	Raw	**0.82 ± 0.38**	0.79 ± 0.27	0.78 ± 0.24	0.77 ± 0.23	**0.77 ± 0.22**
Image	MSE	8	**0.52 ± 0.50**	**0.50 ± 0.32**	**0.48 ± 0.28**	**0.47 ± 0.26**	**0.46 ± 0.25**
Image	MSE	20	0.48 ± 0.50	0.48 ± 0.32	0.48 ± 0.29	0.47 ± 0.27	0.46 ± 0.26
Image	MSE	50	0.49 ± 0.50	0.48 ± 0.32	0.47 ± 0.29	0.46 ± 0.27	0.46 ± 0.26
Image	MSE	100	0.49 ± 0.50	0.48 ± 0.32	0.47 ± 0.29	0.46 ± 0.27	0.46 ± 0.26
Image	SAM	8	0.55 ± 0.50	0.52 ± 0.29	0.51 ± 0.25	0.50 ± 0.22	0.49 ± 0.21
Image	SAM	20	0.57 ± 0.49	0.54 ± 0.31	**0.53 ± 0.25**	**0.51 ± 0.24**	**0.51 ± 0.22**
Image	SAM	50	**0.58 ± 0.49**	**0.54 ± 0.30**	0.52 ± 0.26	**0.51 ± 0.24**	**0.51 ± 0.22**
Image	SAM	100	0.57 ± 0.49	0.54 ± 0.31	0.53 ± 0.26	**0.51 ± 0.24**	**0.51 ± 0.22**
Image	UIQ	8	0.00 ± 0.00	0.00 ± 0.00	0.00 ± 0.00	0.07 ± 0.00	0.10 ± 0.00
Image	UIQ	20	**0.59 ± 0.49**	**0.58 ± 0.35**	**0.58 ± 0.32**	**0.58 ± 0.31**	**0.58 ± 0.30**
Image	UIQ	50	0.55 ± 0.50	0.55 ± 0.33	0.55 ± 0.30	0.54 ± 0.28	0.53 ± 0.27
Image	UIQ	100	0.54 ± 0.50	0.54 ± 0.33	0.52 ± 0.29	0.52 ± 0.27	0.51 ± 0.26
LBP	Cosine	8	0.11 ± 0.32	0.08 ± 0.16	0.08 ± 0.13	0.08 ± 0.12	0.08 ± 0.11
LBP	Cosine	20	0.05 ± 0.22	0.04 ± 0.14	0.04 ± 0.13	0.04 ± 0.12	0.03 ± 0.11
LBP	Cosine	50	**0.59 ± 0.49**	**0.18 ± 0.15**	**0.17 ± 0.15**	**0.21 ± 0.19**	**0.21 ± 0.19**
LBP	Cosine	100	0.50 ± 0.50	0.11 ± 0.11	0.13 ± 0.13	0.14 ± 0.10	0.17 ± 0.13
LBP	Euclidean	8	0.11 ± 0.32	0.08 ± 0.16	0.08 ± 0.13	0.08 ± 0.12	0.08 ± 0.11
LBP	Euclidean	20	0.05 ± 0.22	0.04 ± 0.14	0.04 ± 0.13	0.04 ± 0.12	0.03 ± 0.11
LBP	Euclidean	50	**0.59 ± 0.49**	**0.18 ± 0.15**	**0.17 ± 0.15**	**0.21 ± 0.19**	**0.21 ± 0.19**
LBP	Euclidean	100	0.50 ± 0.50	0.11 ± 0.11	0.13 ± 0.13	0.14 ± 0.10	0.17 ± 0.13
LBP	Jaccard	8	0.11 ± 0.32	0.08 ± 0.16	0.08 ± 0.13	0.08 ± 0.12	0.08 ± 0.11
LBP	Jaccard	20	0.05 ± 0.22	0.04 ± 0.14	0.04 ± 0.13	0.04 ± 0.12	0.03 ± 0.11
LBP	Jaccard	50	**0.59 ± 0.49**	**0.18 ± 0.15**	**0.17 ± 0.15**	**0.21 ± 0.19**	**0.21 ± 0.19**
LBP	Jaccard	100	0.50 ± 0.50	0.11 ± 0.11	0.13 ± 0.13	0.14 ± 0.10	0.17 ± 0.13
LBP	Manhattan	8	0.11 ± 0.32	0.08 ± 0.16	0.08 ± 0.13	0.08 ± 0.12	0.08 ± 0.11
LBP	Manhattan	20	0.05 ± 0.22	0.04 ± 0.14	0.04 ± 0.13	0.04 ± 0.12	0.03 ± 0.11
LBP	Manhattan	50	**0.59 ± 0.49**	**0.18 ± 0.15**	**0.17 ± 0.15**	**0.21 ± 0.19**	**0.21 ± 0.19**
LBP	Manhattan	100	0.50 ± 0.50	0.11 ± 0.11	0.13 ± 0.13	0.14 ± 0.10	0.17 ± 0.13
SIFT	Euclidean	8	0.03 ± 0.18	0.03 ± 0.14	0.03 ± 0.12	0.08 ± 0.08	0.11 ± 0.06
SIFT	Euclidean	20	**0.23 ± 0.42**	**0.22 ± 0.21**	**0.20 ± 0.16**	**0.21 ± 0.14**	**0.21 ± 0.13**
SIFT	Euclidean	50	0.15 ± 0.36	0.15 ± 0.18	0.15 ± 0.13	0.15 ± 0.11	0.14 ± 0.10
SIFT	Euclidean	100	0.12 ± 0.33	0.16 ± 0.18	0.17 ± 0.14	0.17 ± 0.11	0.17 ± 0.10

## Appendix B. ImR Evaluation Results for the Heatsink Dataset

**Table A5:** mAP and std. values. The highest mAP with a relatively lower std. is marked in **bold text**.

Feature	Similarity Metric	Image Size	mAP@1	mAP@5	mAP@10	mAP@15	mAP@20	Average
DefChars	Cosine	Raw	**0.98 ± 0.01**	**0.97 ± 0.02**	**0.97 ± 0.02**	**0.97 ± 0.03**	**0.96 ± 0.03**	**0.97 ± 0.02**
DefChars	Euclidean	Raw	**0.98 ± 0.01**	**0.97 ± 0.02**	**0.97 ± 0.02**	**0.97 ± 0.03**	**0.96 ± 0.03**	**0.97 ± 0.02**
DefChars	Jaccard	Raw	0.64 ± 0.41	0.63 ± 0.43	0.62 ± 0.43	0.61 ± 0.44	0.61 ± 0.44	0.62 ± 0.43
DefChars	Manhattan	Raw	**0.98 ± 0.01**	**0.97 ± 0.02**	0.97 ± 0.03	0.96 ± 0.03	**0.96 ± 0.03**	**0.97 ± 0.02**
Image	MSE	8	**0.89 ± 0.11**	**0.88 ± 0.13**	**0.87 ± 0.13**	**0.87 ± 0.14**	**0.86 ± 0.14**	**0.87 ± 0.13**
Image	MSE	20	0.89 ± 0.13	0.87 ± 0.14	0.87 ± 0.14	0.86 ± 0.15	0.86 ± 0.15	0.87 ± 0.14
Image	MSE	50	0.88 ± 0.13	0.87 ± 0.15	0.86 ± 0.15	0.86 ± 0.15	0.86 ± 0.15	0.87 ± 0.15
Image	MSE	100	0.88 ± 0.13	0.87 ± 0.15	0.86 ± 0.15	0.86 ± 0.15	0.86 ± 0.15	0.87 ± 0.15
Image	SAM	8	**0.84 ± 0.15**	**0.83 ± 0.15**	**0.82 ± 0.16**	**0.82 ± 0.17**	**0.81 ± 0.17**	**0.82 ± 0.16**
Image	SAM	20	0.81 ± 0.24	0.81 ± 0.22	0.82 ± 0.22	0.82 ± 0.21	0.82 ± 0.21	0.82 ± 0.22
Image	SAM	50	0.77 ± 0.30	0.80 ± 0.24	0.81 ± 0.23	0.81 ± 0.23	0.81 ± 0.22	0.80 ± 0.24
Image	SAM	100	0.77 ± 0.29	0.80 ± 0.24	0.81 ± 0.23	0.81 ± 0.23	0.81 ± 0.23	0.80 ± 0.24
Image	UIQ	8	0.50 ± 0.71	0.50 ± 0.14	0.50 ± 0.28	0.50 ± 0.24	0.50 ± 0.21	0.50 ± 0.32
Image	UIQ	20	**0.89 ± 0.10**	0.88 ± 0.10	0.87 ± 0.10	0.87 ± 0.10	0.87 ± 0.10	0.88 ± 0.10
Image	UIQ	50	0.87 ± 0.13	0.88 ± 0.09	0.89 ± 0.07	0.89 ± 0.06	0.89 ± 0.06	**0.88 ± 0.08**
Image	UIQ	100	0.86 ± 0.14	**0.89 ± 0.08**	**0.89 ± 0.06**	**0.89 ± 0.05**	**0.89 ± 0.05**	**0.88 ± 0.08**
LBP	Cosine	8	**0.55 ± 0.41**	**0.53 ± 0.18**	**0.52 ± 0.11**	**0.52 ± 0.11**	**0.52 ± 0.10**	**0.53 ± 0.18**
LBP	Cosine	20	0.39 ± 0.51	0.36 ± 0.31	0.35 ± 0.27	0.34 ± 0.25	0.33 ± 0.24	0.35 ± 0.32
LBP	Cosine	50	0.18 ± 0.16	0.18 ± 0.16	0.19 ± 0.17	0.20 ± 0.18	0.21 ± 0.17	0.19 ± 0.17
LBP	Cosine	100	0.24 ± 0.29	0.24 ± 0.29	0.25 ± 0.31	0.27 ± 0.33	0.27 ± 0.34	0.25 ± 0.31
LBP	Euclidean	8	**0.55 ± 0.41**	**0.53 ± 0.18**	**0.52 ± 0.11**	**0.52 ± 0.11**	**0.52 ± 0.10**	**0.53 ± 0.18**
LBP	Euclidean	20	0.39 ± 0.51	0.36 ± 0.31	0.35 ± 0.27	0.34 ± 0.25	0.33 ± 0.24	0.35 ± 0.32
LBP	Euclidean	50	0.18 ± 0.16	0.18 ± 0.16	0.19 ± 0.17	0.20 ± 0.18	0.21 ± 0.17	0.19 ± 0.17
LBP	Euclidean	100	0.24 ± 0.29	0.24 ± 0.29	0.25 ± 0.31	0.27 ± 0.33	0.27 ± 0.34	0.25 ± 0.31
LBP	Jaccard	8	**0.55 ± 0.41**	**0.53 ± 0.18**	**0.52 ± 0.11**	**0.52 ± 0.11**	**0.52 ± 0.10**	**0.53 ± 0.18**
LBP	Jaccard	20	0.39 ± 0.51	0.36 ± 0.31	0.35 ± 0.27	0.34 ± 0.25	0.33 ± 0.24	0.35 ± 0.32
LBP	Jaccard	50	0.18 ± 0.16	0.18 ± 0.16	0.19 ± 0.17	0.20 ± 0.18	0.21 ± 0.17	0.19 ± 0.17
LBP	Jaccard	100	0.24 ± 0.29	0.24 ± 0.29	0.25 ± 0.31	0.27 ± 0.33	0.27 ± 0.34	0.25 ± 0.31
LBP	Manhattan	8	**0.55 ± 0.41**	**0.53 ± 0.18**	**0.52 ± 0.11**	**0.52 ± 0.11**	**0.52 ± 0.10**	**0.53 ± 0.18**
LBP	Manhattan	20	0.39 ± 0.51	0.36 ± 0.31	0.35 ± 0.27	0.34 ± 0.25	0.33 ± 0.24	0.35 ± 0.32
LBP	Manhattan	50	0.18 ± 0.16	0.18 ± 0.16	0.19 ± 0.17	0.20 ± 0.18	0.21 ± 0.17	0.19 ± 0.17
LBP	Manhattan	100	0.24 ± 0.29	0.24 ± 0.29	0.25 ± 0.31	0.27 ± 0.33	0.27 ± 0.34	0.25 ± 0.31
SIFT	Euclidean	8	**0.51 ± 0.69**	0.50 ± 0.15	0.50 ± 0.29	0.50 ± 0.24	0.50 ± 0.21	0.50 ± 0.32
SIFT	Euclidean	20	0.50 ± 0.69	0.51 ± 0.13	0.51 ± 0.27	0.51 ± 0.23	0.51 ± 0.20	0.51 ± 0.30
SIFT	Euclidean	50	0.49 ± 0.65	0.53 ± 0.09	0.54 ± 0.22	0.53 ± 0.19	0.52 ± 0.17	0.52 ± 0.26
SIFT	Euclidean	100	0.48 ± 0.64	**0.55 ± 0.06**	**0.56 ± 0.18**	**0.55 ± 0.15**	**0.54 ± 0.14**	**0.54 ± 0.23**

**Table A6:** AP and std. values for class 1. The highest AP with a relatively lower std. is marked in **bold text**.

Feature	Similarity Metric	Image Size	AP@1	AP@5	AP@10	AP@15	AP@20
DefChars	Cosine	Raw	**0.98 ± 0.15**	**0.96 ± 0.15**	**0.95 ± 0.15**	**0.95 ± 0.16**	**0.94 ± 0.16**
DefChars	Euclidean	Raw	**0.98 ± 0.15**	**0.96 ± 0.15**	**0.95 ± 0.15**	**0.95 ± 0.16**	**0.94 ± 0.16**
DefChars	Jaccard	Raw	0.35 ± 0.48	0.32 ± 0.26	0.31 ± 0.21	0.31 ± 0.18	0.30 ± 0.17
DefChars	Manhattan	Raw	0.97 ± 0.17	0.96 ± 0.16	0.95 ± 0.16	0.94 ± 0.16	0.94 ± 0.17
Image	MSE	8	**0.81 ± 0.39**	**0.79 ± 0.31**	**0.77 ± 0.30**	**0.77 ± 0.29**	**0.76 ± 0.29**
Image	MSE	20	0.79 ± 0.40	0.77 ± 0.31	0.76 ± 0.30	0.76 ± 0.30	0.75 ± 0.29
Image	MSE	50	0.79 ± 0.41	0.77 ± 0.32	0.76 ± 0.30	0.75 ± 0.30	0.75 ± 0.30
Image	MSE	100	0.79 ± 0.41	0.77 ± 0.32	0.76 ± 0.30	0.76 ± 0.30	0.75 ± 0.30
Image	SAM	8	**0.74 ± 0.44**	**0.72 ± 0.30**	**0.71 ± 0.28**	**0.70 ± 0.27**	**0.69 ± 0.26**
Image	SAM	20	0.64 ± 0.48	0.66 ± 0.30	0.66 ± 0.28	0.67 ± 0.28	0.67 ± 0.27
Image	SAM	50	0.56 ± 0.50	0.64 ± 0.30	0.65 ± 0.29	0.65 ± 0.28	0.65 ± 0.28
Image	SAM	100	0.57 ± 0.50	0.63 ± 0.31	0.65 ± 0.29	0.65 ± 0.28	0.65 ± 0.28
Image	UIQ	8	**1.00 ± 0.02**	0.40 ± 0.01	0.30 ± 0.00	0.33 ± 0.00	0.35 ± 0.00
Image	UIQ	20	0.82 ± 0.39	0.81 ± 0.27	0.80 ± 0.26	0.80 ± 0.25	0.80 ± 0.25
Image	UIQ	50	0.78 ± 0.41	0.82 ± 0.25	0.84 ± 0.23	0.84 ± 0.22	0.85 ± 0.22
Image	UIQ	100	0.76 ± 0.43	**0.83 ± 0.24**	**0.85 ± 0.22**	**0.85 ± 0.21**	**0.86 ± 0.21**
LBP	Cosine	8	0.26 ± 0.44	0.40 ± 0.23	0.44 ± 0.17	0.45 ± 0.15	0.46 ± 0.13
LBP	Cosine	20	**0.75 ± 0.43**	**0.58 ± 0.26**	**0.54 ± 0.27**	**0.52 ± 0.26**	0.50 ± 0.27
LBP	Cosine	50	0.30 ± 0.46	0.29 ± 0.45	0.31 ± 0.43	0.33 ± 0.39	0.33 ± 0.37
LBP	Cosine	100	0.45 ± 0.50	0.44 ± 0.49	0.47 ± 0.46	0.50 ± 0.43	**0.51 ± 0.43**
LBP	Euclidean	8	0.26 ± 0.44	0.40 ± 0.23	0.44 ± 0.17	0.45 ± 0.15	0.46 ± 0.13
LBP	Euclidean	20	**0.75 ± 0.43**	**0.58 ± 0.26**	**0.54 ± 0.27**	**0.52 ± 0.26**	0.50 ± 0.27
LBP	Euclidean	50	0.30 ± 0.46	0.29 ± 0.45	0.31 ± 0.43	0.33 ± 0.39	0.33 ± 0.37
LBP	Euclidean	100	0.45 ± 0.50	0.44 ± 0.49	0.47 ± 0.46	0.50 ± 0.43	**0.51 ± 0.43**
LBP	Jaccard	8	0.26 ± 0.44	0.40 ± 0.23	0.44 ± 0.17	0.45 ± 0.15	0.46 ± 0.13
LBP	Jaccard	20	**0.75 ± 0.43**	**0.58 ± 0.26**	**0.54 ± 0.27**	**0.52 ± 0.26**	0.50 ± 0.27
LBP	Jaccard	50	0.30 ± 0.46	0.29 ± 0.45	0.31 ± 0.43	0.33 ± 0.39	0.33 ± 0.37
LBP	Jaccard	100	0.45 ± 0.50	0.44 ± 0.49	0.47 ± 0.46	0.50 ± 0.43	**0.51 ± 0.43**
LBP	Manhattan	8	0.26 ± 0.44	0.40 ± 0.23	0.44 ± 0.17	0.45 ± 0.15	0.46 ± 0.13
LBP	Manhattan	20	**0.75 ± 0.43**	**0.58 ± 0.26**	**0.54 ± 0.27**	**0.52 ± 0.26**	0.50 ± 0.27
LBP	Manhattan	50	0.30 ± 0.46	0.29 ± 0.45	0.31 ± 0.43	0.33 ± 0.39	0.33 ± 0.37
LBP	Manhattan	100	0.45 ± 0.50	0.44 ± 0.49	0.47 ± 0.46	0.50 ± 0.43	**0.51 ± 0.43**
SIFT	Euclidean	8	**1.00 ± 0.04**	0.40 ± 0.01	0.30 ± 0.00	0.33 ± 0.00	0.35 ± 0.00
SIFT	Euclidean	20	0.99 ± 0.11	0.42 ± 0.10	0.32 ± 0.10	0.35 ± 0.08	0.36 ± 0.07
SIFT	Euclidean	50	0.95 ± 0.22	0.47 ± 0.17	0.38 ± 0.18	0.40 ± 0.15	0.40 ± 0.13
SIFT	Euclidean	100	0.93 ± 0.26	**0.51 ± 0.21**	**0.43 ± 0.22**	**0.45 ± 0.19**	**0.45 ± 0.17**

**Table A7:** AP and std. values for class 2. The highest AP with a relatively lower std. is marked in **bold text**.

Feature	Similarity Metric	Image Size	AP@1	AP@5	AP@10	AP@15	AP@20
DefChars	Cosine	Raw	**0.99 ± 0.10**	**0.99 ± 0.07**	**0.99 ± 0.07**	**0.98 ± 0.08**	**0.98 ± 0.08**
DefChars	Euclidean	Raw	**0.99 ± 0.10**	**0.99 ± 0.07**	**0.99 ± 0.07**	**0.98 ± 0.08**	**0.98 ± 0.08**
DefChars	Jaccard	Raw	0.94 ± 0.25	0.93 ± 0.13	0.92 ± 0.10	0.92 ± 0.09	0.92 ± 0.09
DefChars	Manhattan	Raw	**0.99 ± 0.10**	**0.99 ± 0.07**	0.98 ± 0.08	**0.98 ± 0.08**	**0.98 ± 0.08**
Image	MSE	8	0.97 ± 0.17	0.97 ± 0.13	0.96 ± 0.13	0.96 ± 0.13	0.96 ± 0.13
Image	MSE	20	**0.98 ± 0.15**	**0.97 ± 0.12**	0.97 ± 0.13	**0.97 ± 0.13**	**0.97 ± 0.13**
Image	MSE	50	**0.98 ± 0.15**	**0.97 ± 0.12**	**0.97 ± 0.12**	**0.97 ± 0.13**	**0.97 ± 0.13**
Image	MSE	100	**0.98 ± 0.15**	**0.97 ± 0.12**	**0.97 ± 0.12**	**0.97 ± 0.13**	**0.97 ± 0.13**
Image	SAM	8	0.95 ± 0.22	0.94 ± 0.16	0.94 ± 0.16	0.94 ± 0.15	0.94 ± 0.15
Image	SAM	20	0.98 ± 0.15	0.97 ± 0.12	0.97 ± 0.12	0.97 ± 0.12	0.97 ± 0.12
Image	SAM	50	**0.98 ± 0.14**	**0.97 ± 0.11**	**0.97 ± 0.11**	**0.97 ± 0.11**	**0.97 ± 0.11**
Image	SAM	100	0.98 ± 0.15	**0.97 ± 0.11**	**0.97 ± 0.11**	**0.97 ± 0.11**	**0.97 ± 0.11**
Image	UIQ	8	0.00 ± 0.00	0.60 ± 0.00	0.70 ± 0.00	0.67 ± 0.00	0.65 ± 0.00
Image	UIQ	20	**0.96 ± 0.20**	**0.95 ± 0.17**	**0.94 ± 0.17**	**0.94 ± 0.17**	**0.94 ± 0.17**
Image	UIQ	50	**0.96 ± 0.20**	0.94 ± 0.18	0.94 ± 0.18	0.93 ± 0.18	0.93 ± 0.19
Image	UIQ	100	**0.96 ± 0.20**	0.94 ± 0.18	0.94 ± 0.18	0.93 ± 0.19	0.93 ± 0.19
LBP	Cosine	8	**0.84 ± 0.36**	**0.66 ± 0.22**	**0.60 ± 0.20**	**0.60 ± 0.17**	**0.59 ± 0.16**
LBP	Cosine	20	0.03 ± 0.17	0.14 ± 0.23	0.16 ± 0.23	0.16 ± 0.22	0.16 ± 0.22
LBP	Cosine	50	0.07 ± 0.26	0.07 ± 0.25	0.07 ± 0.25	0.08 ± 0.24	0.09 ± 0.24
LBP	Cosine	100	0.03 ± 0.18	0.03 ± 0.18	0.03 ± 0.17	0.03 ± 0.16	0.03 ± 0.15
LBP	Euclidean	8	**0.84 ± 0.36**	**0.66 ± 0.22**	**0.60 ± 0.20**	**0.60 ± 0.17**	**0.59 ± 0.16**
LBP	Euclidean	20	0.03 ± 0.17	0.14 ± 0.23	0.16 ± 0.23	0.16 ± 0.22	0.16 ± 0.22
LBP	Euclidean	50	0.07 ± 0.26	0.07 ± 0.25	0.07 ± 0.25	0.08 ± 0.24	0.09 ± 0.24
LBP	Euclidean	100	0.03 ± 0.18	0.03 ± 0.18	0.03 ± 0.17	0.03 ± 0.16	0.03 ± 0.15
LBP	Jaccard	8	**0.84 ± 0.36**	**0.66 ± 0.22**	**0.60 ± 0.20**	**0.60 ± 0.17**	**0.59 ± 0.16**
LBP	Jaccard	20	0.03 ± 0.17	0.14 ± 0.23	0.16 ± 0.23	0.16 ± 0.22	0.16 ± 0.22
LBP	Jaccard	50	0.07 ± 0.26	0.07 ± 0.25	0.07 ± 0.25	0.08 ± 0.24	0.09 ± 0.24
LBP	Jaccard	100	0.03 ± 0.18	0.03 ± 0.18	0.03 ± 0.17	0.03 ± 0.16	0.03 ± 0.15
LBP	Manhattan	8	**0.84 ± 0.36**	**0.66 ± 0.22**	**0.60 ± 0.20**	**0.60 ± 0.17**	**0.59 ± 0.16**
LBP	Manhattan	20	0.03 ± 0.17	0.14 ± 0.23	0.16 ± 0.23	0.16 ± 0.22	0.16 ± 0.22
LBP	Manhattan	50	0.07 ± 0.26	0.07 ± 0.25	0.07 ± 0.25	0.08 ± 0.24	0.09 ± 0.24
LBP	Manhattan	100	0.03 ± 0.18	0.03 ± 0.18	0.03 ± 0.17	0.03 ± 0.16	0.03 ± 0.15
SIFT	Euclidean	8	0.02 ± 0.14	**0.61 ± 0.05**	**0.70 ± 0.03**	**0.67 ± 0.03**	**0.65 ± 0.03**
SIFT	Euclidean	20	0.02 ± 0.14	0.60 ± 0.05	0.70 ± 0.04	**0.67 ± 0.03**	**0.65 ± 0.03**
SIFT	Euclidean	50	**0.03 ± 0.16**	0.60 ± 0.07	0.69 ± 0.06	0.66 ± 0.05	0.65 ± 0.04
SIFT	Euclidean	100	0.02 ± 0.15	0.59 ± 0.08	0.69 ± 0.08	0.66 ± 0.06	0.64 ± 0.06

## Appendix C. ImR Evaluation Results for the Lake Ice Dataset

**Table A8:** mAP and std. values. The highest mAP with a relatively lower std. is marked in **bold text**.

Feature	Similarity Metric	Image Size	mAP@1	mAP@5	mAP@10	mAP@15	mAP@20	Average
DefChars	Cosine	Raw	0.95 ± 0.03	0.90 ± 0.06	0.87 ± 0.09	0.85 ± 0.10	0.84 ± 0.12	0.88 ± 0.08
DefChars	Euclidean	Raw	0.95 ± 0.03	0.90 ± 0.07	0.87 ± 0.09	0.85 ± 0.11	0.83 ± 0.13	0.88 ± 0.09
DefChars	Jaccard	Raw	0.84 ± 0.12	0.80 ± 0.17	0.77 ± 0.20	0.74 ± 0.24	0.72 ± 0.27	0.77 ± 0.20
DefChars	Manhattan	Raw	**0.96 ± 0.03**	**0.92 ± 0.05**	**0.89 ± 0.07**	**0.87 ± 0.09**	**0.86 ± 0.11**	**0.90 ± 0.07**
Image	MSE	8	**0.93 ± 0.08**	**0.87 ± 0.14**	**0.82 ± 0.17**	**0.80 ± 0.19**	**0.78 ± 0.21**	**0.84 ± 0.16**
Image	MSE	20	0.93 ± 0.09	0.86 ± 0.16	0.82 ± 0.19	0.79 ± 0.21	0.78 ± 0.23	0.84 ± 0.18
Image	MSE	50	0.93 ± 0.10	0.86 ± 0.16	0.82 ± 0.19	0.79 ± 0.21	0.77 ± 0.23	0.83 ± 0.18
Image	MSE	100	0.93 ± 0.10	0.87 ± 0.16	0.82 ± 0.19	0.80 ± 0.21	0.78 ± 0.23	0.84 ± 0.18
Image	SAM	8	**0.94 ± 0.07**	**0.90 ± 0.09**	**0.85 ± 0.13**	**0.81 ± 0.16**	**0.79 ± 0.18**	**0.86 ± 0.13**
Image	SAM	20	0.94 ± 0.09	0.89 ± 0.13	0.84 ± 0.16	0.81 ± 0.19	0.78 ± 0.21	0.85 ± 0.16
Image	SAM	50	0.93 ± 0.09	0.89 ± 0.12	0.85 ± 0.15	0.81 ± 0.18	0.79 ± 0.20	0.85 ± 0.15
Image	SAM	100	0.93 ± 0.09	0.89 ± 0.12	0.85 ± 0.15	0.81 ± 0.18	0.79 ± 0.20	0.85 ± 0.15
Image	UIQ	8	0.25 ± 0.50	0.25 ± 0.19	0.25 ± 0.26	0.25 ± 0.26	0.25 ± 0.21	0.25 ± 0.28
Image	UIQ	20	0.91 ± 0.12	0.84 ± 0.19	0.79 ± 0.23	0.76 ± 0.26	0.74 ± 0.28	0.81 ± 0.22
Image	UIQ	50	0.92 ± 0.10	0.86 ± 0.17	0.81 ± 0.21	0.78 ± 0.23	0.76 ± 0.26	0.83 ± 0.19
Image	UIQ	100	**0.93 ± 0.10**	**0.86 ± 0.16**	**0.82 ± 0.19**	**0.79 ± 0.22**	**0.77 ± 0.23**	**0.83 ± 0.18**
LBP	Cosine	8	0.13 ± 0.20	0.13 ± 0.18	0.13 ± 0.18	0.13 ± 0.17	0.13 ± 0.18	0.13 ± 0.18
LBP	Cosine	20	0.10 ± 0.10	0.11 ± 0.12	0.11 ± 0.12	0.11 ± 0.12	0.12 ± 0.12	0.11 ± 0.12
LBP	Cosine	50	0.24 ± 0.49	0.20 ± 0.38	0.15 ± 0.27	0.14 ± 0.23	0.13 ± 0.20	0.17 ± 0.31
LBP	Cosine	100	**0.29 ± 0.47**	**0.26 ± 0.42**	**0.27 ± 0.34**	**0.25 ± 0.33**	**0.23 ± 0.31**	**0.26 ± 0.37**
LBP	Euclidean	8	0.13 ± 0.20	0.13 ± 0.18	0.13 ± 0.18	0.13 ± 0.17	0.13 ± 0.18	0.13 ± 0.18
LBP	Euclidean	20	0.10 ± 0.10	0.11 ± 0.12	0.11 ± 0.12	0.11 ± 0.12	0.12 ± 0.12	0.11 ± 0.12
LBP	Euclidean	50	0.24 ± 0.49	0.20 ± 0.38	0.15 ± 0.27	0.14 ± 0.23	0.13 ± 0.20	0.17 ± 0.31
LBP	Euclidean	100	**0.29 ± 0.47**	**0.26 ± 0.42**	**0.27 ± 0.34**	**0.25 ± 0.33**	**0.23 ± 0.31**	**0.26 ± 0.37**
LBP	Jaccard	8	0.13 ± 0.20	0.13 ± 0.18	0.13 ± 0.18	0.13 ± 0.17	0.13 ± 0.18	0.13 ± 0.18
LBP	Jaccard	20	0.10 ± 0.10	0.11 ± 0.12	0.11 ± 0.12	0.11 ± 0.12	0.12 ± 0.12	0.11 ± 0.12
LBP	Jaccard	50	0.24 ± 0.49	0.20 ± 0.38	0.15 ± 0.27	0.14 ± 0.23	0.13 ± 0.20	0.17 ± 0.31
LBP	Jaccard	100	**0.29 ± 0.47**	**0.26 ± 0.42**	**0.27 ± 0.34**	**0.25 ± 0.33**	**0.23 ± 0.31**	**0.26 ± 0.37**
LBP	Manhattan	8	0.13 ± 0.20	0.13 ± 0.18	0.13 ± 0.18	0.13 ± 0.17	0.13 ± 0.18	0.13 ± 0.18
LBP	Manhattan	20	0.10 ± 0.10	0.11 ± 0.12	0.11 ± 0.12	0.11 ± 0.12	0.12 ± 0.12	0.11 ± 0.12
LBP	Manhattan	50	0.24 ± 0.49	0.20 ± 0.38	0.15 ± 0.27	0.14 ± 0.23	0.13 ± 0.20	0.17 ± 0.31
LBP	Manhattan	100	**0.29 ± 0.47**	**0.26 ± 0.42**	**0.27 ± 0.34**	**0.25 ± 0.33**	**0.23 ± 0.31**	**0.26 ± 0.37**
SIFT	Euclidean	8	0.27 ± 0.49	0.26 ± 0.20	0.26 ± 0.27	0.26 ± 0.26	0.26 ± 0.22	0.26 ± 0.29
SIFT	Euclidean	20	0.48 ± 0.38	0.42 ± 0.26	0.42 ± 0.29	0.40 ± 0.29	0.40 ± 0.26	0.42 ± 0.30
SIFT	Euclidean	50	0.61 ± 0.14	0.56 ± 0.14	0.55 ± 0.17	0.53 ± 0.18	0.52 ± 0.18	0.55 ± 0.16
SIFT	Euclidean	100	**0.63 ± 0.16**	**0.65 ± 0.14**	**0.65 ± 0.15**	**0.64 ± 0.15**	**0.62 ± 0.16**	**0.64 ± 0.15**

**Table A9:** AP and std. values for class 1. The highest AP with a relatively lower std. is marked in **bold text**.

Feature	Similarity Metric	Image Size	AP@1	AP@5	AP@10	AP@15	AP@20
DefChars	Cosine	Raw	0.92 ± 0.28	0.87 ± 0.26	0.84 ± 0.27	0.83 ± 0.27	0.83 ± 0.28
DefChars	Euclidean	Raw	0.92 ± 0.27	0.86 ± 0.26	0.84 ± 0.27	0.83 ± 0.27	0.83 ± 0.27
DefChars	Jaccard	Raw	0.80 ± 0.40	0.77 ± 0.27	0.75 ± 0.26	0.72 ± 0.25	0.72 ± 0.24
DefChars	Manhattan	Raw	**0.94 ± 0.24**	**0.88 ± 0.24**	**0.86 ± 0.25**	**0.85 ± 0.26**	**0.84 ± 0.26**
Image	MSE	8	0.94 ± 0.23	0.89 ± 0.24	0.87 ± 0.25	0.86 ± 0.26	0.85 ± 0.25
Image	MSE	20	**0.97 ± 0.18**	**0.91 ± 0.22**	0.88 ± 0.25	**0.87 ± 0.25**	**0.86 ± 0.25**
Image	MSE	50	**0.97 ± 0.18**	**0.91 ± 0.22**	**0.88 ± 0.24**	**0.87 ± 0.25**	**0.86 ± 0.25**
Image	MSE	100	0.96 ± 0.19	**0.91 ± 0.22**	**0.88 ± 0.24**	**0.87 ± 0.25**	**0.86 ± 0.25**
Image	SAM	8	0.96 ± 0.21	0.90 ± 0.22	0.87 ± 0.26	0.85 ± 0.27	0.84 ± 0.28
Image	SAM	20	**0.97 ± 0.17**	0.91 ± 0.21	0.88 ± 0.25	0.86 ± 0.26	**0.85 ± 0.27**
Image	SAM	50	0.97 ± 0.18	0.91 ± 0.21	0.88 ± 0.25	0.86 ± 0.26	**0.85 ± 0.27**
Image	SAM	100	**0.97 ± 0.17**	**0.92 ± 0.21**	**0.89 ± 0.24**	**0.87 ± 0.26**	**0.85 ± 0.27**
Image	UIQ	8	0.00 ± 0.00	0.20 ± 0.01	0.10 ± 0.00	0.07 ± 0.00	0.10 ± 0.00
Image	UIQ	20	**0.96 ± 0.20**	0.89 ± 0.25	0.86 ± 0.26	0.85 ± 0.27	0.84 ± 0.27
Image	UIQ	50	0.95 ± 0.21	**0.89 ± 0.24**	0.87 ± 0.26	0.85 ± 0.27	0.84 ± 0.27
Image	UIQ	100	0.95 ± 0.22	**0.89 ± 0.24**	**0.87 ± 0.25**	**0.86 ± 0.26**	**0.85 ± 0.27**
LBP	Cosine	8	**0.04 ± 0.20**	**0.06 ± 0.17**	0.07 ± 0.16	**0.07 ± 0.16**	**0.07 ± 0.16**
LBP	Cosine	20	0.01 ± 0.09	0.01 ± 0.08	0.01 ± 0.07	0.01 ± 0.07	0.01 ± 0.07
LBP	Cosine	50	0.00 ± 0.00	0.00 ± 0.03	0.01 ± 0.04	0.01 ± 0.04	0.01 ± 0.05
LBP	Cosine	100	0.00 ± 0.00	0.01 ± 0.04	**0.09 ± 0.03**	0.06 ± 0.02	0.05 ± 0.01
LBP	Euclidean	8	**0.04 ± 0.20**	**0.06 ± 0.17**	0.07 ± 0.16	**0.07 ± 0.16**	**0.07 ± 0.16**
LBP	Euclidean	20	0.01 ± 0.09	0.01 ± 0.08	0.01 ± 0.07	0.01 ± 0.07	0.01 ± 0.07
LBP	Euclidean	50	0.00 ± 0.00	0.00 ± 0.03	0.01 ± 0.04	0.01 ± 0.04	0.01 ± 0.05
LBP	Euclidean	100	0.00 ± 0.00	0.01 ± 0.04	**0.09 ± 0.03**	0.06 ± 0.02	0.05 ± 0.01
LBP	Jaccard	8	**0.04 ± 0.20**	**0.06 ± 0.17**	0.07 ± 0.16	**0.07 ± 0.16**	**0.07 ± 0.16**
LBP	Jaccard	20	0.01 ± 0.09	0.01 ± 0.08	0.01 ± 0.07	0.01 ± 0.07	0.01 ± 0.07
LBP	Jaccard	50	0.00 ± 0.00	0.00 ± 0.03	0.01 ± 0.04	0.01 ± 0.04	0.01 ± 0.05
LBP	Jaccard	100	0.00 ± 0.00	0.01 ± 0.04	**0.09 ± 0.03**	0.06 ± 0.02	0.05 ± 0.01
LBP	Manhattan	8	**0.04 ± 0.20**	**0.06 ± 0.17**	0.07 ± 0.16	**0.07 ± 0.16**	**0.07 ± 0.16**
LBP	Manhattan	20	0.01 ± 0.09	0.01 ± 0.08	0.01 ± 0.07	0.01 ± 0.07	0.01 ± 0.07
LBP	Manhattan	50	0.00 ± 0.00	0.00 ± 0.03	0.01 ± 0.04	0.01 ± 0.04	0.01 ± 0.05
LBP	Manhattan	100	0.00 ± 0.00	0.01 ± 0.04	**0.09 ± 0.03**	0.06 ± 0.02	0.05 ± 0.01
SIFT	Euclidean	8	0.00 ± 0.04	0.20 ± 0.01	0.10 ± 0.01	0.07 ± 0.01	0.10 ± 0.00
SIFT	Euclidean	20	0.17 ± 0.38	0.35 ± 0.26	0.29 ± 0.29	0.28 ± 0.31	0.31 ± 0.30
SIFT	Euclidean	50	0.56 ± 0.50	0.61 ± 0.32	0.60 ± 0.30	0.59 ± 0.29	0.58 ± 0.28
SIFT	Euclidean	100	**0.59 ± 0.49**	**0.73 ± 0.29**	**0.74 ± 0.27**	**0.74 ± 0.26**	**0.73 ± 0.25**

**Table A10:** AP and std. values for class 2. The highest AP with a relatively lower std. is marked in **bold text**.

Feature	Similarity Metric	Image Size	AP@1	AP@5	AP@10	AP@15	AP@20
DefChars	Cosine	Raw	0.97 ± 0.17	0.93 ± 0.20	0.91 ± 0.22	0.89 ± 0.23	0.88 ± 0.24
DefChars	Euclidean	Raw	0.97 ± 0.17	0.93 ± 0.19	0.91 ± 0.22	0.90 ± 0.23	0.89 ± 0.24
DefChars	Jaccard	Raw	0.91 ± 0.28	0.91 ± 0.26	0.90 ± 0.27	0.90 ± 0.27	**0.90 ± 0.28**
DefChars	Manhattan	Raw	**0.98 ± 0.14**	**0.95 ± 0.17**	**0.92 ± 0.20**	**0.91 ± 0.21**	**0.90 ± 0.22**
Image	MSE	8	0.97 ± 0.18	0.92 ± 0.22	0.87 ± 0.25	0.83 ± 0.28	0.81 ± 0.29
Image	MSE	20	**0.98 ± 0.14**	**0.93 ± 0.20**	**0.88 ± 0.24**	0.84 ± 0.27	0.82 ± 0.29
Image	MSE	50	0.98 ± 0.15	**0.93 ± 0.20**	**0.88 ± 0.24**	0.84 ± 0.27	0.82 ± 0.29
Image	MSE	100	0.98 ± 0.15	**0.93 ± 0.20**	**0.88 ± 0.24**	**0.85 ± 0.26**	**0.82 ± 0.28**
Image	SAM	8	**0.98 ± 0.13**	0.94 ± 0.19	0.89 ± 0.23	0.85 ± 0.26	0.82 ± 0.28
Image	SAM	20	**0.98 ± 0.13**	0.95 ± 0.18	**0.91 ± 0.22**	0.87 ± 0.25	0.84 ± 0.28
Image	SAM	50	**0.98 ± 0.13**	**0.95 ± 0.17**	**0.91 ± 0.22**	0.87 ± 0.25	**0.84 ± 0.27**
Image	SAM	100	**0.98 ± 0.13**	**0.95 ± 0.17**	**0.91 ± 0.22**	**0.88 ± 0.25**	**0.84 ± 0.27**
Image	UIQ	8	0.00 ± 0.00	0.40 ± 0.01	0.30 ± 0.00	0.40 ± 0.00	0.35 ± 0.00
Image	UIQ	20	0.97 ± 0.17	0.92 ± 0.21	0.87 ± 0.25	0.84 ± 0.27	0.81 ± 0.29
Image	UIQ	50	**0.98 ± 0.16**	**0.93 ± 0.20**	**0.88 ± 0.24**	**0.85 ± 0.27**	**0.82 ± 0.29**
Image	UIQ	100	**0.98 ± 0.16**	**0.93 ± 0.20**	**0.88 ± 0.24**	0.84 ± 0.27	**0.82 ± 0.29**
LBP	Cosine	8	0.02 ± 0.12	0.02 ± 0.09	0.03 ± 0.08	0.03 ± 0.08	0.04 ± 0.08
LBP	Cosine	20	**0.23 ± 0.42**	0.11 ± 0.15	0.12 ± 0.13	0.14 ± 0.13	0.15 ± 0.13
LBP	Cosine	50	0.00 ± 0.00	0.01 ± 0.04	0.06 ± 0.11	0.07 ± 0.13	0.08 ± 0.14
LBP	Cosine	100	0.18 ± 0.39	**0.16 ± 0.18**	**0.23 ± 0.13**	**0.22 ± 0.16**	**0.21 ± 0.17**
LBP	Euclidean	8	0.02 ± 0.12	0.02 ± 0.09	0.03 ± 0.08	0.03 ± 0.08	0.04 ± 0.08
LBP	Euclidean	20	**0.23 ± 0.42**	0.11 ± 0.15	0.12 ± 0.13	0.14 ± 0.13	0.15 ± 0.13
LBP	Euclidean	50	0.00 ± 0.00	0.01 ± 0.04	0.06 ± 0.11	0.07 ± 0.13	0.08 ± 0.14
LBP	Euclidean	100	0.18 ± 0.39	**0.16 ± 0.18**	**0.23 ± 0.13**	**0.22 ± 0.16**	**0.21 ± 0.17**
LBP	Jaccard	8	0.02 ± 0.12	0.02 ± 0.09	0.03 ± 0.08	0.03 ± 0.08	0.04 ± 0.08
LBP	Jaccard	20	**0.23 ± 0.42**	0.11 ± 0.15	0.12 ± 0.13	0.14 ± 0.13	0.15 ± 0.13
LBP	Jaccard	50	0.00 ± 0.00	0.01 ± 0.04	0.06 ± 0.11	0.07 ± 0.13	0.08 ± 0.14
LBP	Jaccard	100	0.18 ± 0.39	**0.16 ± 0.18**	**0.23 ± 0.13**	**0.22 ± 0.16**	**0.21 ± 0.17**
LBP	Manhattan	8	0.02 ± 0.12	0.02 ± 0.09	0.03 ± 0.08	0.03 ± 0.08	0.04 ± 0.08
LBP	Manhattan	20	**0.23 ± 0.42**	0.11 ± 0.15	0.12 ± 0.13	0.14 ± 0.13	0.15 ± 0.13
LBP	Manhattan	50	0.00 ± 0.00	0.01 ± 0.04	0.06 ± 0.11	0.07 ± 0.13	0.08 ± 0.14
LBP	Manhattan	100	0.18 ± 0.39	**0.16 ± 0.18**	**0.23 ± 0.13**	**0.22 ± 0.16**	**0.21 ± 0.17**
SIFT	Euclidean	8	0.07 ± 0.26	0.44 ± 0.15	0.34 ± 0.16	0.43 ± 0.12	0.38 ± 0.11
SIFT	Euclidean	20	**0.68 ± 0.47**	**0.72 ± 0.28**	0.69 ± 0.30	**0.69 ± 0.27**	0.67 ± 0.28
SIFT	Euclidean	50	0.63 ± 0.48	0.60 ± 0.34	0.57 ± 0.32	0.57 ± 0.29	0.56 ± 0.28
SIFT	Euclidean	100	0.66 ± 0.47	0.70 ± 0.30	**0.70 ± 0.28**	**0.69 ± 0.27**	**0.68 ± 0.27**

**Table A11:** AP and std. values for class 3. The highest AP with a relatively lower std. is marked in **bold text**.

Feature	Similarity Metric	Image Size	AP@1	AP@5	AP@10	AP@15	AP@20
DefChars	Cosine	Raw	0.99 ± 0.12	0.98 ± 0.11	0.97 ± 0.12	0.97 ± 0.12	0.96 ± 0.13
DefChars	Euclidean	Raw	0.99 ± 0.12	0.98 ± 0.11	0.97 ± 0.12	0.96 ± 0.13	0.96 ± 0.13
DefChars	Jaccard	Raw	0.97 ± 0.18	0.95 ± 0.16	0.94 ± 0.16	0.93 ± 0.16	0.92 ± 0.16
DefChars	Manhattan	Raw	**0.99 ± 0.10**	**0.98 ± 0.09**	**0.98 ± 0.10**	**0.97 ± 0.11**	**0.97 ± 0.11**
Image	MSE	8	0.99 ± 0.10	0.98 ± 0.09	0.98 ± 0.10	**0.98 ± 0.10**	0.97 ± 0.10
Image	MSE	20	0.99 ± 0.10	**0.99 ± 0.09**	**0.98 ± 0.09**	**0.98 ± 0.10**	**0.98 ± 0.10**
Image	MSE	50	**0.99 ± 0.09**	0.98 ± 0.09	**0.98 ± 0.09**	**0.98 ± 0.10**	**0.98 ± 0.10**
Image	MSE	100	0.99 ± 0.10	0.98 ± 0.09	**0.98 ± 0.09**	**0.98 ± 0.10**	**0.98 ± 0.10**
Image	SAM	8	**0.99 ± 0.10**	**0.98 ± 0.10**	**0.97 ± 0.11**	0.96 ± 0.12	**0.96 ± 0.12**
Image	SAM	20	**0.99 ± 0.10**	**0.98 ± 0.10**	**0.97 ± 0.11**	**0.97 ± 0.12**	**0.96 ± 0.12**
Image	SAM	50	0.99 ± 0.11	0.98 ± 0.11	0.97 ± 0.12	0.96 ± 0.13	0.96 ± 0.13
Image	SAM	100	0.99 ± 0.11	0.98 ± 0.11	0.97 ± 0.12	0.96 ± 0.13	0.96 ± 0.13
Image	UIQ	8	**1.00 ± 0.02**	0.40 ± 0.00	0.60 ± 0.00	0.53 ± 0.00	0.50 ± 0.00
Image	UIQ	20	0.99 ± 0.09	0.98 ± 0.09	0.98 ± 0.10	0.97 ± 0.10	0.97 ± 0.11
Image	UIQ	50	0.99 ± 0.08	**0.99 ± 0.07**	0.98 ± 0.08	**0.98 ± 0.08**	**0.98 ± 0.08**
Image	UIQ	100	0.99 ± 0.07	0.99 ± 0.08	**0.99 ± 0.08**	**0.98 ± 0.08**	**0.98 ± 0.08**
LBP	Cosine	8	0.43 ± 0.49	0.40 ± 0.31	0.39 ± 0.26	0.39 ± 0.24	0.39 ± 0.23
LBP	Cosine	20	0.13 ± 0.33	0.27 ± 0.31	0.28 ± 0.25	0.27 ± 0.21	0.27 ± 0.19
LBP	Cosine	50	0.97 ± 0.16	0.77 ± 0.17	0.56 ± 0.15	0.48 ± 0.16	0.42 ± 0.16
LBP	Cosine	100	**0.99 ± 0.11**	**0.87 ± 0.19**	**0.76 ± 0.16**	**0.72 ± 0.14**	**0.68 ± 0.12**
LBP	Euclidean	8	0.43 ± 0.49	0.40 ± 0.31	0.39 ± 0.26	0.39 ± 0.24	0.39 ± 0.23
LBP	Euclidean	20	0.13 ± 0.33	0.27 ± 0.31	0.28 ± 0.25	0.27 ± 0.21	0.27 ± 0.19
LBP	Euclidean	50	0.97 ± 0.16	0.77 ± 0.17	0.56 ± 0.15	0.48 ± 0.16	0.42 ± 0.16
LBP	Euclidean	100	**0.99 ± 0.11**	**0.87 ± 0.19**	**0.76 ± 0.16**	**0.72 ± 0.14**	**0.68 ± 0.12**
LBP	Jaccard	8	0.43 ± 0.49	0.40 ± 0.31	0.39 ± 0.26	0.39 ± 0.24	0.39 ± 0.23
LBP	Jaccard	20	0.13 ± 0.33	0.27 ± 0.31	0.28 ± 0.25	0.27 ± 0.21	0.27 ± 0.19
LBP	Jaccard	50	0.97 ± 0.16	0.77 ± 0.17	0.56 ± 0.15	0.48 ± 0.16	0.42 ± 0.16
LBP	Jaccard	100	**0.99 ± 0.11**	**0.87 ± 0.19**	**0.76 ± 0.16**	**0.72 ± 0.14**	**0.68 ± 0.12**
LBP	Manhattan	8	0.43 ± 0.49	0.40 ± 0.31	0.39 ± 0.26	0.39 ± 0.24	0.39 ± 0.23
LBP	Manhattan	20	0.13 ± 0.33	0.27 ± 0.31	0.28 ± 0.25	0.27 ± 0.21	0.27 ± 0.19
LBP	Manhattan	50	0.97 ± 0.16	0.77 ± 0.17	0.56 ± 0.15	0.48 ± 0.16	0.42 ± 0.16
LBP	Manhattan	100	**0.99 ± 0.11**	**0.87 ± 0.19**	**0.76 ± 0.16**	**0.72 ± 0.14**	**0.68 ± 0.12**
SIFT	Euclidean	8	**1.00 ± 0.05**	0.40 ± 0.05	0.60 ± 0.03	0.54 ± 0.02	0.50 ± 0.02
SIFT	Euclidean	20	0.93 ± 0.26	0.50 ± 0.22	0.62 ± 0.15	0.57 ± 0.15	0.55 ± 0.15
SIFT	Euclidean	50	0.79 ± 0.41	0.68 ± 0.29	0.71 ± 0.23	0.70 ± 0.22	0.69 ± 0.22
SIFT	Euclidean	100	0.83 ± 0.38	**0.74 ± 0.27**	**0.73 ± 0.23**	**0.71 ± 0.22**	**0.70 ± 0.21**

**Table A12:** AP and std. values for class 4. The highest AP with a relatively lower std. is marked in **bold text**.

Feature	Similarity Metric	Image Size	AP@1	AP@5	AP@10	AP@15	AP@20
DefChars	Cosine	Raw	0.93 ± 0.25	0.85 ± 0.26	0.77 ± 0.28	0.72 ± 0.29	0.68 ± 0.29
DefChars	Euclidean	Raw	0.92 ± 0.27	0.83 ± 0.27	0.76 ± 0.28	0.71 ± 0.29	0.66 ± 0.30
DefChars	Jaccard	Raw	0.69 ± 0.46	0.58 ± 0.40	0.50 ± 0.38	0.40 ± 0.29	0.33 ± 0.23
DefChars	Manhattan	Raw	**0.94 ± 0.23**	**0.89 ± 0.23**	**0.81 ± 0.25**	**0.76 ± 0.27**	**0.72 ± 0.27**
Image	MSE	8	**0.81 ± 0.40**	**0.67 ± 0.36**	**0.58 ± 0.35**	**0.52 ± 0.33**	**0.47 ± 0.31**
Image	MSE	20	0.79 ± 0.41	0.63 ± 0.39	0.54 ± 0.37	0.49 ± 0.34	0.45 ± 0.32
Image	MSE	50	0.78 ± 0.41	0.63 ± 0.38	0.54 ± 0.37	0.49 ± 0.35	0.44 ± 0.33
Image	MSE	100	0.78 ± 0.41	0.63 ± 0.39	0.54 ± 0.37	0.49 ± 0.35	0.44 ± 0.33
Image	SAM	8	**0.84 ± 0.36**	**0.76 ± 0.35**	**0.66 ± 0.35**	**0.59 ± 0.33**	**0.53 ± 0.32**
Image	SAM	20	0.80 ± 0.40	0.70 ± 0.40	0.61 ± 0.39	0.54 ± 0.38	0.49 ± 0.37
Image	SAM	50	0.80 ± 0.40	0.71 ± 0.40	0.62 ± 0.38	0.55 ± 0.37	0.50 ± 0.37
Image	SAM	100	0.80 ± 0.40	0.71 ± 0.40	0.62 ± 0.38	0.55 ± 0.37	0.50 ± 0.37
Image	UIQ	8	0.00 ± 0.00	0.00 ± 0.00	0.00 ± 0.00	0.00 ± 0.00	0.05 ± 0.00
Image	UIQ	20	0.74 ± 0.44	0.57 ± 0.40	0.45 ± 0.35	0.38 ± 0.30	0.33 ± 0.26
Image	UIQ	50	0.77 ± 0.42	0.61 ± 0.40	0.51 ± 0.37	0.45 ± 0.34	0.39 ± 0.30
Image	UIQ	100	**0.78 ± 0.41**	**0.63 ± 0.39**	**0.54 ± 0.37**	**0.48 ± 0.36**	**0.44 ± 0.33**
LBP	Cosine	8	0.03 ± 0.17	0.02 ± 0.08	0.02 ± 0.07	0.02 ± 0.06	0.02 ± 0.06
LBP	Cosine	20	**0.04 ± 0.20**	**0.04 ± 0.11**	**0.04 ± 0.10**	**0.04 ± 0.09**	**0.04 ± 0.09**
LBP	Cosine	50	0.00 ± 0.00	0.00 ± 0.00	0.00 ± 0.00	0.00 ± 0.01	0.00 ± 0.01
LBP	Cosine	100	0.00 ± 0.00	0.00 ± 0.00	0.00 ± 0.00	0.00 ± 0.00	0.00 ± 0.00
LBP	Euclidean	8	0.03 ± 0.17	0.02 ± 0.08	0.02 ± 0.07	0.02 ± 0.06	0.02 ± 0.06
LBP	Euclidean	20	**0.04 ± 0.20**	**0.04 ± 0.11**	**0.04 ± 0.10**	**0.04 ± 0.09**	**0.04 ± 0.09**
LBP	Euclidean	50	0.00 ± 0.00	0.00 ± 0.00	0.00 ± 0.00	0.00 ± 0.01	0.00 ± 0.01
LBP	Euclidean	100	0.00 ± 0.00	0.00 ± 0.00	0.00 ± 0.00	0.00 ± 0.00	0.00 ± 0.00
LBP	Jaccard	8	0.03 ± 0.17	0.02 ± 0.08	0.02 ± 0.07	0.02 ± 0.06	0.02 ± 0.06
LBP	Jaccard	20	**0.04 ± 0.20**	**0.04 ± 0.11**	**0.04 ± 0.10**	**0.04 ± 0.09**	**0.04 ± 0.09**
LBP	Jaccard	50	0.00 ± 0.00	0.00 ± 0.00	0.00 ± 0.00	0.00 ± 0.01	0.00 ± 0.01
LBP	Jaccard	100	0.00 ± 0.00	0.00 ± 0.00	0.00 ± 0.00	0.00 ± 0.00	0.00 ± 0.00
LBP	Manhattan	8	0.03 ± 0.17	0.02 ± 0.08	0.02 ± 0.07	0.02 ± 0.06	0.02 ± 0.06
LBP	Manhattan	20	**0.04 ± 0.20**	**0.04 ± 0.11**	**0.04 ± 0.10**	**0.04 ± 0.09**	**0.04 ± 0.09**
LBP	Manhattan	50	0.00 ± 0.00	0.00 ± 0.00	0.00 ± 0.00	0.00 ± 0.01	0.00 ± 0.01
LBP	Manhattan	100	0.00 ± 0.00	0.00 ± 0.00	0.00 ± 0.00	0.00 ± 0.00	0.00 ± 0.00
SIFT	Euclidean	8	0.00 ± 0.00	0.00 ± 0.00	0.00 ± 0.00	0.00 ± 0.00	0.05 ± 0.00
SIFT	Euclidean	20	0.15 ± 0.36	0.10 ± 0.20	0.07 ± 0.14	0.06 ± 0.12	0.09 ± 0.09
SIFT	Euclidean	50	**0.46 ± 0.50**	0.36 ± 0.32	0.31 ± 0.27	0.28 ± 0.25	0.27 ± 0.22
SIFT	Euclidean	100	0.43 ± 0.50	**0.45 ± 0.35**	**0.44 ± 0.31**	**0.41 ± 0.28**	**0.39 ± 0.26**

## Appendix D. ImR Evaluation Results for the Wind Turbine Blade Dataset

**Table A13:** mAP and std. values. The highest mAP with a relatively lower std. is marked in **bold text**.

Feature	Similarity Metric	Image Size	mAP@1	mAP@5	mAP@10	mAP@15	mAP@20	Average
DefChars	Cosine	Raw	0.74 ± 0.06	0.62 ± 0.14	0.57 ± 0.16	0.54 ± 0.18	0.52 ± 0.19	0.60 ± 0.15
DefChars	Euclidean	Raw	0.75 ± 0.08	0.63 ± 0.15	0.58 ± 0.17	0.54 ± 0.19	0.52 ± 0.20	0.60 ± 0.16
DefChars	Jaccard	Raw	0.41 ± 0.20	0.41 ± 0.17	0.37 ± 0.18	0.36 ± 0.17	0.36 ± 0.17	0.38 ± 0.18
DefChars	Manhattan	Raw	**0.77 ± 0.09**	**0.64 ± 0.17**	**0.59 ± 0.18**	**0.55 ± 0.20**	**0.53 ± 0.20**	**0.62 ± 0.17**
Image	MSE	8	0.52 ± 0.23	**0.44 ± 0.33**	0.42 ± 0.34	**0.42 ± 0.34**	**0.40 ± 0.33**	**0.44 ± 0.31**
Image	MSE	20	0.54 ± 0.28	0.44 ± 0.35	**0.43 ± 0.36**	0.41 ± 0.35	0.40 ± 0.35	0.44 ± 0.34
Image	MSE	50	**0.54 ± 0.27**	0.44 ± 0.36	**0.43 ± 0.36**	0.41 ± 0.35	0.40 ± 0.35	0.44 ± 0.34
Image	MSE	100	**0.54 ± 0.27**	0.44 ± 0.35	0.42 ± 0.36	0.41 ± 0.35	0.40 ± 0.35	0.44 ± 0.34
Image	SAM	8	**0.52 ± 0.20**	0.41 ± 0.30	**0.41 ± 0.32**	0.39 ± 0.33	0.38 ± 0.32	0.42 ± 0.29
Image	SAM	20	0.51 ± 0.22	**0.43 ± 0.33**	0.41 ± 0.34	0.39 ± 0.35	**0.39 ± 0.35**	**0.43 ± 0.32**
Image	SAM	50	0.50 ± 0.25	0.42 ± 0.34	0.41 ± 0.35	0.39 ± 0.35	0.38 ± 0.35	0.42 ± 0.33
Image	SAM	100	0.51 ± 0.25	**0.43 ± 0.33**	0.41 ± 0.35	**0.40 ± 0.35**	0.38 ± 0.35	0.43 ± 0.33
Image	UIQ	8	0.25 ± 0.50	0.25 ± 0.19	0.25 ± 0.10	0.25 ± 0.10	0.25 ± 0.07	0.25 ± 0.19
Image	UIQ	20	0.50 ± 0.28	0.43 ± 0.33	0.42 ± 0.32	**0.42 ± 0.32**	**0.41 ± 0.31**	**0.44 ± 0.31**
Image	UIQ	50	0.50 ± 0.27	0.44 ± 0.34	**0.43 ± 0.34**	0.42 ± 0.33	0.41 ± 0.32	0.44 ± 0.32
Image	UIQ	100	**0.52 ± 0.25**	**0.45 ± 0.35**	0.42 ± 0.35	0.42 ± 0.33	0.41 ± 0.32	0.44 ± 0.32
LBP	Cosine	8	**0.26 ± 0.23**	**0.28 ± 0.14**	**0.26 ± 0.16**	**0.26 ± 0.16**	**0.25 ± 0.15**	**0.26 ± 0.17**
LBP	Cosine	20	0.19 ± 0.25	0.20 ± 0.29	0.20 ± 0.28	0.21 ± 0.25	0.21 ± 0.21	0.20 ± 0.26
LBP	Cosine	50	0.17 ± 0.28	0.17 ± 0.27	0.16 ± 0.25	0.17 ± 0.23	0.17 ± 0.24	0.17 ± 0.25
LBP	Cosine	100	0.16 ± 0.26	0.18 ± 0.29	0.17 ± 0.29	0.18 ± 0.27	0.18 ± 0.28	0.17 ± 0.28
LBP	Euclidean	8	**0.26 ± 0.23**	**0.28 ± 0.14**	**0.26 ± 0.16**	**0.26 ± 0.16**	**0.25 ± 0.15**	**0.26 ± 0.17**
LBP	Euclidean	20	0.19 ± 0.25	0.20 ± 0.29	0.20 ± 0.28	0.21 ± 0.25	0.21 ± 0.21	0.20 ± 0.26
LBP	Euclidean	50	0.17 ± 0.28	0.17 ± 0.27	0.16 ± 0.25	0.17 ± 0.23	0.17 ± 0.24	0.17 ± 0.25
LBP	Euclidean	100	0.16 ± 0.26	0.18 ± 0.29	0.17 ± 0.29	0.18 ± 0.27	0.18 ± 0.28	0.17 ± 0.28
LBP	Jaccard	8	**0.26 ± 0.23**	**0.28 ± 0.14**	**0.26 ± 0.16**	**0.26 ± 0.16**	**0.25 ± 0.15**	**0.26 ± 0.17**
LBP	Jaccard	20	0.19 ± 0.25	0.20 ± 0.29	0.20 ± 0.28	0.21 ± 0.25	0.21 ± 0.21	0.20 ± 0.26
LBP	Jaccard	50	0.17 ± 0.28	0.17 ± 0.27	0.16 ± 0.25	0.17 ± 0.23	0.17 ± 0.24	0.17 ± 0.25
LBP	Jaccard	100	0.16 ± 0.26	0.18 ± 0.29	0.17 ± 0.29	0.18 ± 0.27	0.18 ± 0.28	0.17 ± 0.28
LBP	Manhattan	8	**0.26 ± 0.23**	**0.28 ± 0.14**	**0.26 ± 0.16**	**0.26 ± 0.16**	**0.25 ± 0.15**	**0.26 ± 0.17**
LBP	Manhattan	20	0.19 ± 0.25	0.20 ± 0.29	0.20 ± 0.28	0.21 ± 0.25	0.21 ± 0.21	0.20 ± 0.26
LBP	Manhattan	50	0.17 ± 0.28	0.17 ± 0.27	0.16 ± 0.25	0.17 ± 0.23	0.17 ± 0.24	0.17 ± 0.25
LBP	Manhattan	100	0.16 ± 0.26	0.18 ± 0.29	0.17 ± 0.29	0.18 ± 0.27	0.18 ± 0.28	0.17 ± 0.28
SIFT	Euclidean	8	0.24 ± 0.47	0.26 ± 0.21	0.26 ± 0.11	0.25 ± 0.11	0.25 ± 0.08	0.25 ± 0.20
SIFT	Euclidean	20	0.30 ± 0.36	0.30 ± 0.26	0.32 ± 0.22	0.31 ± 0.22	0.30 ± 0.21	0.31 ± 0.25
SIFT	Euclidean	50	0.38 ± 0.20	**0.34 ± 0.24**	**0.34 ± 0.22**	0.32 ± 0.22	0.32 ± 0.22	0.34 ± 0.22
SIFT	Euclidean	100	**0.40 ± 0.31**	0.34 ± 0.33	0.33 ± 0.31	**0.34 ± 0.27**	**0.33 ± 0.26**	**0.35 ± 0.30**

**Table A14:** AP and std. values for class 1. The highest AP with a relatively lower std. is marked in **bold text**.

Feature	Similarity Metric	Image Size	AP@1	AP@5	AP@10	AP@15	AP@20
DefChars	Cosine	Raw	0.75 ± 0.43	0.64 ± 0.30	0.59 ± 0.28	**0.55 ± 0.26**	0.52 ± 0.25
DefChars	Euclidean	Raw	0.73 ± 0.45	0.64 ± 0.30	**0.59 ± 0.26**	**0.55 ± 0.26**	**0.53 ± 0.24**
DefChars	Jaccard	Raw	0.31 ± 0.47	0.37 ± 0.23	0.31 ± 0.16	0.30 ± 0.14	0.29 ± 0.12
DefChars	Manhattan	Raw	**0.78 ± 0.42**	**0.64 ± 0.29**	0.59 ± 0.27	**0.55 ± 0.26**	**0.53 ± 0.24**
Image	MSE	8	**0.71 ± 0.46**	0.70 ± 0.36	0.68 ± 0.31	0.67 ± 0.29	0.65 ± 0.28
Image	MSE	20	**0.71 ± 0.46**	0.71 ± 0.36	**0.73 ± 0.30**	0.70 ± 0.30	0.69 ± 0.28
Image	MSE	50	0.70 ± 0.46	0.72 ± 0.36	**0.73 ± 0.30**	0.71 ± 0.29	0.69 ± 0.28
Image	MSE	100	0.70 ± 0.46	**0.73 ± 0.35**	**0.73 ± 0.30**	**0.71 ± 0.28**	**0.69 ± 0.27**
Image	SAM	8	**0.74 ± 0.44**	0.69 ± 0.35	0.71 ± 0.31	0.70 ± 0.30	0.68 ± 0.27
Image	SAM	20	0.71 ± 0.46	0.74 ± 0.35	**0.75 ± 0.30**	0.74 ± 0.29	0.74 ± 0.27
Image	SAM	50	0.72 ± 0.45	**0.74 ± 0.34**	**0.75 ± 0.30**	**0.74 ± 0.28**	**0.74 ± 0.26**
Image	SAM	100	0.73 ± 0.45	**0.74 ± 0.34**	**0.75 ± 0.30**	**0.74 ± 0.28**	0.73 ± 0.26
Image	UIQ	8	0.00 ± 0.00	0.00 ± 0.00	0.10 ± 0.00	0.20 ± 0.00	0.20 ± 0.01
Image	UIQ	20	0.67 ± 0.47	0.69 ± 0.36	0.70 ± 0.32	0.71 ± 0.29	0.69 ± 0.26
Image	UIQ	50	**0.69 ± 0.47**	0.73 ± 0.34	0.74 ± 0.30	0.73 ± 0.27	0.72 ± 0.25
Image	UIQ	100	**0.69 ± 0.47**	**0.76 ± 0.33**	**0.76 ± 0.28**	**0.75 ± 0.26**	**0.73 ± 0.24**
LBP	Cosine	8	**0.56 ± 0.50**	0.31 ± 0.16	0.23 ± 0.10	0.22 ± 0.10	0.22 ± 0.10
LBP	Cosine	20	0.52 ± 0.50	**0.63 ± 0.38**	**0.62 ± 0.32**	**0.56 ± 0.28**	**0.49 ± 0.23**
LBP	Cosine	50	0.10 ± 0.30	0.12 ± 0.26	0.12 ± 0.24	0.18 ± 0.20	0.16 ± 0.17
LBP	Cosine	100	0.11 ± 0.32	0.10 ± 0.22	0.08 ± 0.17	0.13 ± 0.15	0.12 ± 0.15
LBP	Euclidean	8	**0.56 ± 0.50**	0.31 ± 0.16	0.23 ± 0.10	0.22 ± 0.10	0.22 ± 0.10
LBP	Euclidean	20	0.52 ± 0.50	**0.63 ± 0.38**	**0.62 ± 0.32**	**0.56 ± 0.28**	**0.49 ± 0.23**
LBP	Euclidean	50	0.10 ± 0.30	0.12 ± 0.26	0.12 ± 0.24	0.18 ± 0.20	0.16 ± 0.17
LBP	Euclidean	100	0.11 ± 0.32	0.10 ± 0.22	0.08 ± 0.17	0.13 ± 0.15	0.12 ± 0.15
LBP	Jaccard	8	**0.56 ± 0.50**	0.31 ± 0.16	0.23 ± 0.10	0.22 ± 0.10	0.22 ± 0.10
LBP	Jaccard	20	0.52 ± 0.50	**0.63 ± 0.38**	**0.62 ± 0.32**	**0.56 ± 0.28**	**0.49 ± 0.23**
LBP	Jaccard	50	0.10 ± 0.30	0.12 ± 0.26	0.12 ± 0.24	0.18 ± 0.20	0.16 ± 0.17
LBP	Jaccard	100	0.11 ± 0.32	0.10 ± 0.22	0.08 ± 0.17	0.13 ± 0.15	0.12 ± 0.15
LBP	Manhattan	8	**0.56 ± 0.50**	0.31 ± 0.16	0.23 ± 0.10	0.22 ± 0.10	0.22 ± 0.10
LBP	Manhattan	20	0.52 ± 0.50	**0.63 ± 0.38**	**0.62 ± 0.32**	**0.56 ± 0.28**	**0.49 ± 0.23**
LBP	Manhattan	50	0.10 ± 0.30	0.12 ± 0.26	0.12 ± 0.24	0.18 ± 0.20	0.16 ± 0.17
LBP	Manhattan	100	0.11 ± 0.32	0.10 ± 0.22	0.08 ± 0.17	0.13 ± 0.15	0.12 ± 0.15
SIFT	Euclidean	8	0.00 ± 0.00	0.01 ± 0.04	0.10 ± 0.01	0.19 ± 0.03	0.20 ± 0.01
SIFT	Euclidean	20	0.21 ± 0.41	0.13 ± 0.18	0.16 ± 0.12	0.21 ± 0.09	0.22 ± 0.07
SIFT	Euclidean	50	0.35 ± 0.48	**0.33 ± 0.26**	**0.30 ± 0.19**	**0.29 ± 0.16**	**0.28 ± 0.12**
SIFT	Euclidean	100	**0.31 ± 0.47**	0.22 ± 0.19	0.26 ± 0.16	0.27 ± 0.13	0.27 ± 0.11

**Table A15:** AP and std. values for class 2. The highest AP with a relatively lower std. is marked in **bold text**.

Feature	Similarity Metric	Image Size	AP@1	AP@5	AP@10	AP@15	AP@20
DefChars	Cosine	Raw	0.68 ± 0.47	0.65 ± 0.32	0.62 ± 0.27	**0.62 ± 0.25**	**0.61 ± 0.24**
DefChars	Euclidean	Raw	0.68 ± 0.47	0.64 ± 0.32	0.62 ± 0.28	0.59 ± 0.27	0.58 ± 0.26
DefChars	Jaccard	Raw	0.45 ± 0.50	0.42 ± 0.28	0.41 ± 0.20	0.41 ± 0.18	0.41 ± 0.17
DefChars	Manhattan	Raw	**0.70 ± 0.46**	**0.66 ± 0.30**	**0.63 ± 0.29**	0.60 ± 0.28	0.59 ± 0.27
Image	MSE	8	0.41 ± 0.50	**0.25 ± 0.21**	**0.23 ± 0.17**	**0.21 ± 0.13**	**0.20 ± 0.12**
Image	MSE	20	0.42 ± 0.50	0.23 ± 0.20	0.20 ± 0.16	0.19 ± 0.14	0.17 ± 0.12
Image	MSE	50	**0.44 ± 0.50**	0.22 ± 0.20	0.20 ± 0.15	0.18 ± 0.13	0.17 ± 0.11
Image	MSE	100	**0.44 ± 0.50**	0.23 ± 0.20	0.20 ± 0.15	0.18 ± 0.13	0.17 ± 0.11
Image	SAM	8	**0.37 ± 0.49**	**0.21 ± 0.22**	**0.19 ± 0.19**	**0.16 ± 0.15**	**0.15 ± 0.12**
Image	SAM	20	0.30 ± 0.46	0.18 ± 0.23	0.15 ± 0.19	0.13 ± 0.16	0.13 ± 0.13
Image	SAM	50	0.32 ± 0.47	0.17 ± 0.21	0.16 ± 0.19	0.14 ± 0.16	0.13 ± 0.12
Image	SAM	100	0.30 ± 0.46	0.18 ± 0.22	0.16 ± 0.19	0.15 ± 0.16	0.13 ± 0.12
Image	UIQ	8	0.00 ± 0.00	**0.39 ± 0.03**	**0.30 ± 0.02**	0.20 ± 0.01	0.20 ± 0.01
Image	UIQ	20	0.32 ± 0.47	0.24 ± 0.24	0.24 ± 0.20	0.22 ± 0.16	0.22 ± 0.14
Image	UIQ	50	0.34 ± 0.48	0.25 ± 0.24	0.25 ± 0.19	**0.23 ± 0.15**	0.22 ± 0.14
Image	UIQ	100	**0.42 ± 0.50**	0.26 ± 0.24	0.23 ± 0.18	0.23 ± 0.16	**0.22 ± 0.13**
LBP	Cosine	8	**0.27 ± 0.45**	**0.36 ± 0.18**	**0.39 ± 0.16**	**0.37 ± 0.14**	**0.36 ± 0.14**
LBP	Cosine	20	0.00 ± 0.00	0.01 ± 0.05	0.04 ± 0.08	0.06 ± 0.08	0.10 ± 0.08
LBP	Cosine	50	0.00 ± 0.00	0.00 ± 0.02	0.00 ± 0.02	0.00 ± 0.02	0.01 ± 0.04
LBP	Cosine	100	0.00 ± 0.00	0.00 ± 0.00	0.00 ± 0.00	0.00 ± 0.00	0.00 ± 0.00
LBP	Euclidean	8	**0.27 ± 0.45**	**0.36 ± 0.18**	**0.39 ± 0.16**	**0.37 ± 0.14**	**0.36 ± 0.14**
LBP	Euclidean	20	0.00 ± 0.00	0.01 ± 0.05	0.04 ± 0.08	0.06 ± 0.08	0.10 ± 0.08
LBP	Euclidean	50	0.00 ± 0.00	0.00 ± 0.02	0.00 ± 0.02	0.00 ± 0.02	0.01 ± 0.04
LBP	Euclidean	100	0.00 ± 0.00	0.00 ± 0.00	0.00 ± 0.00	0.00 ± 0.00	0.00 ± 0.00
LBP	Jaccard	8	**0.27 ± 0.45**	**0.36 ± 0.18**	**0.39 ± 0.16**	**0.37 ± 0.14**	**0.36 ± 0.14**
LBP	Jaccard	20	0.00 ± 0.00	0.01 ± 0.05	0.04 ± 0.08	0.06 ± 0.08	0.10 ± 0.08
LBP	Jaccard	50	0.00 ± 0.00	0.00 ± 0.02	0.00 ± 0.02	0.00 ± 0.02	0.01 ± 0.04
LBP	Jaccard	100	0.00 ± 0.00	0.00 ± 0.00	0.00 ± 0.00	0.00 ± 0.00	0.00 ± 0.00
LBP	Manhattan	8	**0.27 ± 0.45**	**0.36 ± 0.18**	**0.39 ± 0.16**	**0.37 ± 0.14**	**0.36 ± 0.14**
LBP	Manhattan	20	0.00 ± 0.00	0.01 ± 0.05	0.04 ± 0.08	0.06 ± 0.08	0.10 ± 0.08
LBP	Manhattan	50	0.00 ± 0.00	0.00 ± 0.02	0.00 ± 0.02	0.00 ± 0.02	0.01 ± 0.04
LBP	Manhattan	100	0.00 ± 0.00	0.00 ± 0.00	0.00 ± 0.00	0.00 ± 0.00	0.00 ± 0.00
SIFT	Euclidean	8	0.00 ± 0.00	**0.39 ± 0.04**	**0.30 ± 0.02**	0.20 ± 0.01	0.20 ± 0.01
SIFT	Euclidean	20	0.08 ± 0.28	0.29 ± 0.16	0.27 ± 0.11	0.21 ± 0.07	0.19 ± 0.05
SIFT	Euclidean	50	**0.26 ± 0.44**	0.21 ± 0.21	0.25 ± 0.15	**0.23 ± 0.11**	0.21 ± 0.08
SIFT	Euclidean	100	0.21 ± 0.41	0.18 ± 0.21	0.19 ± 0.15	0.22 ± 0.15	**0.22 ± 0.12**

**Table A16:** AP and std. values for class 3. The highest AP with a relatively lower std. is marked in **bold text**.

Feature	Similarity Metric	Image Size	AP@1	AP@5	AP@10	AP@15	AP@20
DefChars	Cosine	Raw	0.82 ± 0.38	0.77 ± 0.27	0.73 ± 0.27	0.71 ± 0.27	0.69 ± 0.26
DefChars	Euclidean	Raw	0.87 ± 0.33	0.79 ± 0.26	0.75 ± 0.27	0.74 ± 0.26	0.72 ± 0.24
DefChars	Jaccard	Raw	0.68 ± 0.47	0.64 ± 0.30	0.60 ± 0.23	0.58 ± 0.20	0.56 ± 0.19
DefChars	Manhattan	Raw	**0.90 ± 0.30**	**0.83 ± 0.25**	**0.78 ± 0.26**	**0.76 ± 0.25**	**0.74 ± 0.25**
Image	MSE	8	0.70 ± 0.46	0.74 ± 0.29	**0.74 ± 0.27**	**0.73 ± 0.25**	**0.71 ± 0.25**
Image	MSE	20	**0.82 ± 0.38**	**0.76 ± 0.29**	0.74 ± 0.28	0.72 ± 0.26	**0.71 ± 0.25**
Image	MSE	50	**0.82 ± 0.38**	**0.76 ± 0.29**	0.74 ± 0.28	0.72 ± 0.27	0.71 ± 0.26
Image	MSE	100	**0.82 ± 0.38**	0.75 ± 0.29	0.74 ± 0.28	0.72 ± 0.27	0.71 ± 0.26
Image	SAM	8	0.64 ± 0.48	0.65 ± 0.31	0.64 ± 0.30	**0.64 ± 0.28**	0.62 ± 0.27
Image	SAM	20	0.70 ± 0.46	0.67 ± 0.33	0.65 ± 0.31	0.64 ± 0.30	0.63 ± 0.30
Image	SAM	50	**0.71 ± 0.45**	**0.68 ± 0.33**	**0.66 ± 0.31**	0.64 ± 0.31	**0.63 ± 0.29**
Image	SAM	100	**0.71 ± 0.45**	0.68 ± 0.34	0.65 ± 0.31	0.64 ± 0.31	0.63 ± 0.30
Image	UIQ	8	**1.00 ± 0.00**	0.40 ± 0.00	0.30 ± 0.02	0.40 ± 0.01	0.35 ± 0.00
Image	UIQ	20	0.81 ± 0.40	**0.73 ± 0.30**	**0.69 ± 0.28**	**0.68 ± 0.27**	**0.66 ± 0.27**
Image	UIQ	50	0.78 ± 0.42	0.72 ± 0.31	0.68 ± 0.28	0.65 ± 0.28	0.64 ± 0.27
Image	UIQ	100	0.75 ± 0.44	0.71 ± 0.32	0.67 ± 0.30	0.65 ± 0.28	0.63 ± 0.27
LBP	Cosine	8	0.22 ± 0.42	0.36 ± 0.27	0.37 ± 0.19	0.38 ± 0.16	0.38 ± 0.14
LBP	Cosine	20	0.25 ± 0.44	0.18 ± 0.27	0.16 ± 0.20	0.20 ± 0.16	0.21 ± 0.14
LBP	Cosine	50	**0.58 ± 0.50**	0.57 ± 0.36	0.53 ± 0.38	0.50 ± 0.35	0.50 ± 0.33
LBP	Cosine	100	0.54 ± 0.50	**0.61 ± 0.39**	**0.61 ± 0.39**	**0.57 ± 0.35**	**0.59 ± 0.33**
LBP	Euclidean	8	0.22 ± 0.42	0.36 ± 0.27	0.37 ± 0.19	0.38 ± 0.16	0.38 ± 0.14
LBP	Euclidean	20	0.25 ± 0.44	0.18 ± 0.27	0.16 ± 0.20	0.20 ± 0.16	0.21 ± 0.14
LBP	Euclidean	50	**0.58 ± 0.50**	0.57 ± 0.36	0.53 ± 0.38	0.50 ± 0.35	0.50 ± 0.33
LBP	Euclidean	100	0.54 ± 0.50	**0.61 ± 0.39**	**0.61 ± 0.39**	**0.57 ± 0.35**	**0.59 ± 0.33**
LBP	Jaccard	8	0.22 ± 0.42	0.36 ± 0.27	0.37 ± 0.19	0.38 ± 0.16	0.38 ± 0.14
LBP	Jaccard	20	0.25 ± 0.44	0.18 ± 0.27	0.16 ± 0.20	0.20 ± 0.16	0.21 ± 0.14
LBP	Jaccard	50	**0.58 ± 0.50**	0.57 ± 0.36	0.53 ± 0.38	0.50 ± 0.35	0.50 ± 0.33
LBP	Jaccard	100	0.54 ± 0.50	**0.61 ± 0.39**	**0.61 ± 0.39**	**0.57 ± 0.35**	**0.59 ± 0.33**
LBP	Manhattan	8	0.22 ± 0.42	0.36 ± 0.27	0.37 ± 0.19	0.38 ± 0.16	0.38 ± 0.14
LBP	Manhattan	20	0.25 ± 0.44	0.18 ± 0.27	0.16 ± 0.20	0.20 ± 0.16	0.21 ± 0.14
LBP	Manhattan	50	**0.58 ± 0.50**	0.57 ± 0.36	0.53 ± 0.38	0.50 ± 0.35	0.50 ± 0.33
LBP	Manhattan	100	0.54 ± 0.50	**0.61 ± 0.39**	**0.61 ± 0.39**	**0.57 ± 0.35**	**0.59 ± 0.33**
SIFT	Euclidean	8	**0.95 ± 0.22**	0.46 ± 0.14	0.34 ± 0.10	0.41 ± 0.03	0.37 ± 0.04
SIFT	Euclidean	20	0.83 ± 0.38	0.67 ± 0.24	0.64 ± 0.26	0.64 ± 0.21	0.61 ± 0.22
SIFT	Euclidean	50	0.67 ± 0.47	0.69 ± 0.22	0.66 ± 0.19	0.64 ± 0.17	0.63 ± 0.15
SIFT	Euclidean	100	0.86 ± 0.34	**0.83 ± 0.21**	**0.78 ± 0.20**	**0.73 ± 0.19**	**0.70 ± 0.17**

**Table A17:** AP and std. values for class 4. The highest AP with a relatively lower std. is marked in **bold text**.

Feature	Similarity Metric	Image Size	AP@1	AP@5	AP@10	AP@15	AP@20
DefChars	Cosine	Raw	**0.71 ± 0.46**	0.43 ± 0.38	0.35 ± 0.28	**0.30 ± 0.20**	0.25 ± 0.16
DefChars	Euclidean	Raw	**0.71 ± 0.46**	**0.43 ± 0.34**	**0.35 ± 0.27**	0.29 ± 0.19	0.24 ± 0.15
DefChars	Jaccard	Raw	0.21 ± 0.41	0.23 ± 0.22	0.17 ± 0.13	0.17 ± 0.11	0.17 ± 0.08
DefChars	Manhattan	Raw	**0.71 ± 0.46**	0.42 ± 0.35	0.34 ± 0.24	0.29 ± 0.18	**0.26 ± 0.14**
Image	MSE	8	**0.25 ± 0.44**	**0.08 ± 0.13**	**0.05 ± 0.07**	**0.05 ± 0.05**	**0.04 ± 0.04**
Image	MSE	20	0.21 ± 0.41	0.06 ± 0.09	**0.05 ± 0.07**	0.04 ± 0.05	**0.04 ± 0.04**
Image	MSE	50	0.21 ± 0.41	0.05 ± 0.09	0.03 ± 0.06	0.04 ± 0.05	**0.04 ± 0.04**
Image	MSE	100	0.21 ± 0.41	0.05 ± 0.09	0.03 ± 0.06	0.04 ± 0.05	**0.04 ± 0.04**
Image	SAM	8	**0.33 ± 0.48**	**0.11 ± 0.14**	**0.08 ± 0.09**	**0.06 ± 0.07**	**0.06 ± 0.06**
Image	SAM	20	**0.33 ± 0.48**	0.11 ± 0.20	0.08 ± 0.11	**0.06 ± 0.07**	0.05 ± 0.06
Image	SAM	50	0.25 ± 0.44	0.10 ± 0.19	0.07 ± 0.11	0.05 ± 0.07	0.04 ± 0.06
Image	SAM	100	0.29 ± 0.46	0.10 ± 0.19	0.06 ± 0.11	0.05 ± 0.07	0.05 ± 0.06
Image	UIQ	8	0.00 ± 0.00	**0.19 ± 0.04**	**0.29 ± 0.03**	**0.19 ± 0.02**	**0.24 ± 0.02**
Image	UIQ	20	**0.21 ± 0.41**	0.08 ± 0.12	0.05 ± 0.08	0.06 ± 0.09	0.06 ± 0.07
Image	UIQ	50	**0.21 ± 0.41**	0.05 ± 0.09	0.05 ± 0.06	0.05 ± 0.05	0.05 ± 0.04
Image	UIQ	100	**0.21 ± 0.41**	0.05 ± 0.09	0.03 ± 0.05	0.05 ± 0.05	0.05 ± 0.04
LBP	Cosine	8	**0.00 ± 0.00**	**0.07 ± 0.10**	**0.05 ± 0.07**	**0.05 ± 0.06**	**0.05 ± 0.05**
LBP	Cosine	20	**0.00 ± 0.00**	0.00 ± 0.00	0.00 ± 0.00	0.01 ± 0.03	0.01 ± 0.03
LBP	Cosine	50	**0.00 ± 0.00**	0.00 ± 0.00	0.00 ± 0.00	0.00 ± 0.00	0.00 ± 0.00
LBP	Cosine	100	**0.00 ± 0.00**	0.00 ± 0.00	0.00 ± 0.00	0.00 ± 0.00	0.00 ± 0.00
LBP	Euclidean	8	**0.00 ± 0.00**	**0.07 ± 0.10**	**0.05 ± 0.07**	**0.05 ± 0.06**	**0.05 ± 0.05**
LBP	Euclidean	20	**0.00 ± 0.00**	0.00 ± 0.00	0.00 ± 0.00	0.01 ± 0.03	0.01 ± 0.03
LBP	Euclidean	50	**0.00 ± 0.00**	0.00 ± 0.00	0.00 ± 0.00	0.00 ± 0.00	0.00 ± 0.00
LBP	Euclidean	100	**0.00 ± 0.00**	0.00 ± 0.00	0.00 ± 0.00	0.00 ± 0.00	0.00 ± 0.00
LBP	Jaccard	8	**0.00 ± 0.00**	**0.07 ± 0.10**	**0.05 ± 0.07**	**0.05 ± 0.06**	**0.05 ± 0.05**
LBP	Jaccard	20	**0.00 ± 0.00**	0.00 ± 0.00	0.00 ± 0.00	0.01 ± 0.03	0.01 ± 0.03
LBP	Jaccard	50	**0.00 ± 0.00**	0.00 ± 0.00	0.00 ± 0.00	0.00 ± 0.00	0.00 ± 0.00
LBP	Jaccard	100	**0.00 ± 0.00**	0.00 ± 0.00	0.00 ± 0.00	0.00 ± 0.00	0.00 ± 0.00
LBP	Manhattan	8	**0.00 ± 0.00**	**0.07 ± 0.10**	**0.05 ± 0.07**	**0.05 ± 0.06**	**0.05 ± 0.05**
LBP	Manhattan	20	**0.00 ± 0.00**	0.00 ± 0.00	0.00 ± 0.00	0.01 ± 0.03	0.01 ± 0.03
LBP	Manhattan	50	**0.00 ± 0.00**	0.00 ± 0.00	0.00 ± 0.00	0.00 ± 0.00	0.00 ± 0.00
LBP	Manhattan	100	**0.00 ± 0.00**	0.00 ± 0.00	0.00 ± 0.00	0.00 ± 0.00	0.00 ± 0.00
SIFT	Euclidean	8	0.00 ± 0.00	**0.19 ± 0.04**	**0.29 ± 0.03**	**0.19 ± 0.02**	**0.24 ± 0.02**
SIFT	Euclidean	20	0.08 ± 0.28	0.13 ± 0.13	0.21 ± 0.12	0.18 ± 0.07	0.18 ± 0.05
SIFT	Euclidean	50	**0.25 ± 0.44**	0.14 ± 0.16	0.15 ± 0.10	0.14 ± 0.07	0.14 ± 0.06
SIFT	Euclidean	100	0.21 ± 0.41	0.13 ± 0.18	0.10 ± 0.12	0.13 ± 0.09	0.13 ± 0.07

## References

[1] Halawani A., Teynor A., Setia L., Brunner G., Burkhardt H. (2006). Fundamentals and Applications of Image Retrieval: An Overview. Datenbank-Spektrum.

[2] Nakazawa T., Kulkarni D.V. (2018). Wafer Map Defect Pattern Classification and Image Retrieval Using Convolutional Neural Network. IEEE Trans. Semicond. Manuf..

[3] Hu X., Fu M., Zhu Z., Xiang Z., Qian M., Wang J. (2021). Unsupervised defect detection algorithm for printed fabrics using content-based image retrieval techniques. Text. Res. J..

[4] Liu P., El-Gohary N. Semantic Image Retrieval and Clustering for Supporting Domain-Specific Bridge Component and Defect Classification. Proceedings of the Construction Research Congress 2020.

[5] Agrawal S., Chowdhary A., Agarwala S., Mayya V., Kamath S.S. (2022). Content-based medical image retrieval system for lung diseases using deep CNNs. Int. J. Inf. Technol..

[6] Xie B., Zhuang Y., Jiang N., Liu J. An effective and efficient framework of content-based similarity retrieval of large CT image sequences based on WSLEN model. Multimed. Tools Appl..

[7] Choe J., Hwang H.J., Seo J.B., Lee S.M., Yun J., Kim M.J., Jeong J., Lee Y., Jin K., Park R. (2022). Content-based Image Retrieval by Using Deep Learning for Interstitial Lung Disease Diagnosis with Chest CT. Radiology.

[8] Scott K.A., Xu L., Pour H.K. (2020). Retrieval of ice/water observations from synthetic aperture radar imagery for use in lake ice data assimilation. J. Great Lakes Res..

[9] Stonevicius E., Uselis G., Grendaite D. (2022). Ice Detection with Sentinel-1 SAR Backscatter Threshold in Long Sections of Temperate Climate Rivers. Remote Sens..

[10] Wang Z., Bovik A. (2002). A universal image quality index. IEEE Signal Process. Lett..

[11] Yuhas R.H., Goetz A.F., Boardman J.W. Discrimination among semi-arid landscape endmembers using the spectral angle mapper (SAM) algorithm. Proceedings of the JPL, Summaries of the Third Annual JPL Airborne Geoscience Workshop, Volume 1: AVIRIS Workshop.

[12] VenkatNarayanaRao T., Govardhan A. (2013). Assessment of Diverse Quality Metrics for Medical Images Including Mammography. Int. J. Comput. Appl..

[13] Wang Z., Bovik A., Sheikh H., Simoncelli E. (2004). Image quality assessment: From error visibility to structural similarity. IEEE Trans. Image Process..

[14] Rajith B., Srivastava M., Agarwal S., Shetty N.R., Prasad N., Nalini N. (2016). Edge Preserved De-noising Method for Medical X-Ray Images Using Wavelet Packet Transformation. Emerging Research in Computing, Information, Communication and Applications.

[15] Zhang Y. (2008). Methods for image fusion quality assessment—A review, comparison and analysis. Int. Arch. Photogramm. Remote Sens. Spat. Inf. Sci..

[16] Boudani F.Z., Nacereddine N., Laiche N., Hernández Heredia Y., Milián Núñez V., Ruiz Shulcloper J. (2021). Content-Based Image Retrieval for Surface Defects of Hot Rolled Steel Strip Using Wavelet-Based LBP. Progress in Artificial Intelligence and Pattern Recognition.

[17] Zhang L., Liu X., Lu Z., Liu F., Hong R. (2015). Lace Fabric Image Retrieval Based on Multi-Scale and Rotation Invariant LBP. Proceedings of the 7th International Conference on Internet Multimedia Computing and Service, ICIMCS ’15.

[18] Khan A., Rajvee M.H., Deekshatulu B.L., Pratap Reddy L., Chakravarthy V., Bhateja V., Flores Fuentes W., Anguera J., Vasavi K.P. (2023). A Fused LBP Texture Descriptor-Based Image Retrieval System. Advances in Signal Processing, Embedded Systems and IoT.

[19] Lai W.C., Srividhya S.R. (2022). A Modified LBP Operator-Based Optimized Fuzzy Art Map Medical Image Retrieval System for Disease Diagnosis and Prediction. Biomedicines.

[20] Zhi L.J., Zhang S.M., Zhao D.Z., Zhao H., Lin S.K. Medical Image Retrieval Using SIFT Feature. Proceedings of the 2009 2nd International Congress on Image and Signal Processing.

[21] Cruz B.F., de Assis J.T., Estrela V.V., Khelassi A. (2019). A Compact Sift-Based Strategy for Visual Information Retrieval in Large Image Databases: Array. Med. Technol. J..

[22] Srinivas M., Naidu R.R., Sastry C., Mohan C.K. (2015). Content based medical image retrieval using dictionary learning. Neurocomputing.

[23] Patel B., Yadav k., Ghosh D. State-of-Art: Similarity Assessment for Content Based Image Retrieval System. Proceedings of the 2020 IEEE International Symposium on Sustainable Energy, Signal Processing and Cyber Security (iSSSC).

[24] Seetharaman K., Sathiamoorthy S. (2016). A unified learning framework for content based medical image retrieval using a statistical model. J. King Saud Univ.-Comput. Inf. Sci..

[25] Schettini R., Ciocca G., Gagliardi I. (2009). Feature Extraction for Content-Based Image Retrieval. Encyclopedia of Database Systems.

[26] Yuan Z.W., Zhang J., Falco C.M., Jiang X. (2016). Feature extraction and image retrieval based on AlexNet. Proceedings of the Eighth International Conference on Digital Image Processing (ICDIP 2016).

[27] Ali A., Sharma S. Content based image retrieval using feature extraction with machine learning. Proceedings of the 2017 International Conference on Intelligent Computing and Control Systems (ICICCS).

[28] Piras L., Giacinto G. (2017). Information fusion in content based image retrieval: A comprehensive overview. Inf. Fusion.

[29] Zhang J., Cosma G., Bugby S., Finke A., Watkins J. (2023). Morphological Image Analysis and Feature Extraction for Reasoning with AI-based Defect Detection and Classification Models. arXiv.

[30] Zhang J., Cosma G., Bugby S., Watkins J. (2023). ForestMonkey: Toolkit for Reasoning with AI-based Defect Detection and Classification Models. arXiv.

[31] Zhang J., Cosma G., Watkins J. (2021). Image Enhanced Mask R-CNN: A Deep Learning Pipeline with New Evaluation Measures for Wind Turbine Blade Defect Detection and Classification. J. Imaging.

[32] Ter-Sarkisov A. (2022). COVID-CT-Mask-Net: Prediction of COVID-19 from CT Scans Using Regional Features. Appl. Intell..

[33] Yang K., Liu Y., Zhang S., Cao J. (2022). Surface Defect Detection of Heat Sink Based on Lightweight Fully Convolutional Network. IEEE Trans. Instrum. Meas..

[34] Prabha R., Tom M., Rothermel M., Baltsavias E., Leal-Taixe L., Schindler K. (2020). Lake ice monitoring with webcams and crowd-sourced images. ISPRS Ann. Photogramm. Remote Sens. Spat. Inf. Sci..

[35] Latif A., Rasheed A., Sajid U., Ahmed J., Ali N., Ratyal N.I., Zafar B., Dar S.H., Sajid M., Khalil T. (2019). Content-Based Image Retrieval and Feature Extraction: A Comprehensive Review. Math. Probl. Eng..

[36] Shao H., Wu Y., Cui W., Zhang J. Image Retrieval Based on MPEG-7 Dominant Color Descriptor. Proceedings of the 2008 The 9th International Conference for Young Computer Scientists.

[37] Duanmu X. Image Retrieval Using Color Moment Invariant. Proceedings of the 2010 Seventh International Conference on Information Technology: New Generations.

[38] Wang X.Y., Zhang B.B., Yang H.Y. (2014). Content-based image retrieval by integrating color and texture features. Multimed. Tools Appl..

[39] Liu Y., Zhang D., Lu G. (2008). Region-based image retrieval with high-level semantics using decision tree learning. Pattern Recognit..

[40] Zhang H., Dong Z., Shu H. Object recognition by a complete set of pseudo-Zernike moment invariants. Proceedings of the 2010 IEEE International Conference on Acoustics, Speech and Signal Processing.

[41] Guo J.M., Prasetyo H., Chen J.H. (2015). Content-Based Image Retrieval Using Error Diffusion Block Truncation Coding Features. IEEE Trans. Circuits Syst. Video Technol..

[42] Jiexian Z., Xiupeng L., Yu F. (2014). Multiscale Distance Coherence Vector Algorithm for Content-Based Image Retrieval. Sci. World J..

[43] Islam M.M., Zhang D., Lu G. Automatic Categorization of Image Regions Using Dominant Color Based Vector Quantization. Proceedings of the 2008 Digital Image Computing: Techniques and Applications.

[44] Papakostas G., Koulouriotis D., Tourassis V., Chessa M., Solari F., Sabatini S.P. (2012). Feature Extraction Based on Wavelet Moments and Moment Invariants in Machine Vision Systems. Human-Centric Machine Vision.

[45] Liu G.H., Li Z.Y., Zhang L., Xu Y. (2011). Image retrieval based on micro-structure descriptor. Pattern Recognit..

[46] Wang X.Y., Chen Z.F., Yun J.J. (2012). An effective method for color image retrieval based on texture. Comput. Stand. Interfaces.

[47] Ashraf R., Bashir K., Irtaza A., Mahmood M.T. (2015). Content Based Image Retrieval Using Embedded Neural Networks with Bandletized Regions. Entropy.

[48] Irtaza A., Jaffar M.A. (2015). Categorical image retrieval through genetically optimized support vector machines (GOSVM) and hybrid texture features. Signal Image Video Process..

[49] Fadaei S., Amirfattahi R., Ahmadzadeh M.R. (2017). Local derivative radial patterns: A new texture descriptor for content-based image retrieval. Signal Process..

[50] Wang X., Wang Z. (2013). A novel method for image retrieval based on structure elements’ descriptor. J. Vis. Commun. Image Represent..

[51] Ali N., Bajwa K.B., Sablatnig R., Chatzichristofis S.A., Iqbal Z., Rashid M., Habib H.A. (2016). A Novel Image Retrieval Based on Visual Words Integration of SIFT and SURF. PLoS ONE.

[52] Lazebnik S., Schmid C., Ponce J. Beyond Bags of Features: Spatial Pyramid Matching for Recognizing Natural Scene Categories. Proceedings of the 2006 IEEE Computer Society Conference on Computer Vision and Pattern Recognition (CVPR’06).

[53] Mehmood Z., Anwar S.M., Ali N., Habib H.A., Rashid M. (2016). A Novel Image Retrieval Based on a Combination of Local and Global Histograms of Visual Words. Math. Probl. Eng..

[54] Naeem M., Ashraf R., Ali N., Ahmad M., Habib M.A. (2017). Bottom up Approach for Better Requirements Elicitation. Proceedings of the International Conference on Future Networks and Distributed Systems (ICFNDS ’17).

[55] Zafar B., Ashraf R., Ali N., Iqbal M., Sajid M., Dar S., Ratyal N. (2018). A Novel Discriminating and Relative Global Spatial Image Representation with Applications in CBIR. Appl. Sci..

[56] Anwar H., Zambanini S., Kampel M. A rotation-invariant bag of visual words model for symbols based ancient coin classification. Proceedings of the 2014 IEEE International Conference on Image Processing (ICIP).

[57] Khan R., Barat C., Muselet D., Ducottet C. (2015). Spatial histograms of soft pairwise similar patches to improve the bag-of-visual-words model. Comput. Vis. Image Underst..

[58] Ashraf R., Ahmed M., Ahmad U., Habib M.A., Jabbar S., Naseer K. (2020). MDCBIR-MF: Multimedia data for content-based image retrieval by using multiple features. Multimed. Tools Appl..

[59] Mistry Y., Ingole D., Ingole M. (2018). Content based image retrieval using hybrid features and various distance metric. J. Electr. Syst. Inf. Technol..

[60] Ahmed K.T., Ummesafi S., Iqbal A. (2019). Content based image retrieval using image features information fusion. Inf. Fusion.

[61] Liu P., Guo J.M., Chamnongthai K., Prasetyo H. (2017). Fusion of color histogram and LBP-based features for texture image retrieval and classification. Inf. Sci..

[62] Nazir A., Ashraf R., Hamdani T., Ali N. Content based image retrieval system by using HSV color histogram, discrete wavelet transform and edge histogram descriptor. Proceedings of the 2018 International Conference on Computing, Mathematics and Engineering Technologies (iCoMET).

[63] Kang L.W., Hsu C.Y., Chen H.W., Lu C.S., Lin C.Y., Pei S.C. (2011). Feature-Based Sparse Representation for Image Similarity Assessment. IEEE Trans. Multimed..

[64] Zhao Z.Q., Glotin H., Xie Z., Gao J., Wu X. (2012). Cooperative Sparse Representation in Two Opposite Directions for Semi-Supervised Image Annotation. IEEE Trans. Image Process..

[65] Thiagarajan J.J., Natesan Ramamurthy K., Sattigeri P., Spanias A. Supervised local sparse coding of sub-image features for image retrieval. Proceedings of the 2012 19th IEEE International Conference on Image Processing.

[66] Wang D., Hoi S.C., He Y., Zhu J. (2011). Retrieval-Based Face Annotation by Weak Label Regularized Local Coordinate Coding. Proceedings of the 19th ACM International Conference on Multimedia (MM ’11).

[67] Hong C., Zhu J. (2013). Hypergraph-based multi-example ranking with sparse representation for transductive learning image retrieval. Neurocomputing.

[68] Mohamadzadeh S., Farsi H. (2016). Content-based image retrieval system via sparse representation. IET Comput. Vis..

[69] Li Q., Han Y., Dang J. (2016). Sketch4Image: A novel framework for sketch-based image retrieval based on product quantization with coding residuals. Multimed. Tools Appl..

[70] Duan Y., Lu J., Feng J., Zhou J. (2018). Context-Aware Local Binary Feature Learning for Face Recognition. IEEE Trans. Pattern Anal. Mach. Intell..

[71] Shamna P., Govindan V., Abdul Nazeer K. (2022). Content-based medical image retrieval by spatial matching of visual words. J. King Saud Univ.-Comput. Inf. Sci..

[72] Mo D., Wong W.K., Liu X., Ge Y. (2022). Concentrated hashing with neighborhood embedding for image retrieval and classification. Int. J. Mach. Learn. Cybern..

[73] Deep G., Kaur J., Singh S.P., Nayak S.R., Kumar M., Kautish S. (2022). MeQryEP: A Texture Based Descriptor for Biomedical Image Retrieval. J. Healthc. Eng..

[74] Tan R.Z., Venkatarayalu N., Atmosukarto I., Premkumar A.B., Teh T.E., Thinn K.K., Xue M. Supervised Image Retrieval and Ranking Technique for Lock-in Thermography Images. Proceedings of the 2022 IEEE International Symposium on the Physical and Failure Analysis of Integrated Circuits (IPFA).

[75] Gassner M., Barranco Garcia J., Tanadini-Lang S., Bertoldo F., Fröhlich F., Guckenberger M., Haueis S., Pelzer C., Reyes M., Schmithausen P. (2023). Saliency-Enhanced Content-Based Image Retrieval for Diagnosis Support in Dermatology Consultation: Reader Study. JMIR Dermatol..

[76] Chen W., Liu Y., Wang W., Bakker E.M., Georgiou T., Fieguth P., Liu L., Lew M.S. (2023). Deep Learning for Instance Retrieval: A Survey. IEEE Trans. Pattern Anal. Mach. Intell..

[77] Dutta A., Zisserman A. (2019). The VIA Annotation Software for Images, Audio and Video. Proceedings of the 27th ACM International Conference on Multimedia (MM ’19).

[78] Russell B.C., Torralba A., Murphy K.P., Freeman W.T. (2008). LabelMe: A Database and Web-Based Tool for Image Annotation. Int. J. Comput. Vis..

[79] Bradski G. (2000). The OpenCV Library. Dr. Dobb’s J. Softw. Tools.

[80] Lowe D.G. (2004). Distinctive Image Features from Scale-Invariant Keypoints. Int. J. Comput. Vis..

[81] Ojala T., Pietikainen M., Harwood D. Performance evaluation of texture measures with classification based on Kullback discrimination of distributions. Proceedings of the 12th International Conference on Pattern Recognition.

[82] Rahillda Nadhirah N.R., Hashim U.R., Salahuddin L., Kanchymalay K., Aji P.W., Teo H.C. (2022). Local Texture Representation for Timber Defect Recognition based on Variation of LBP. Int. J. Adv. Comput. Sci. Appl..

[83] Shao S., Chen K., Karpur A., Cui Q., Araujo A., Cao B. Global Features are All You Need for Image Retrieval and Reranking. Proceedings of the IEEE/CVF International Conference on Computer Vision (ICCV).

[84] Radenović F., Iscen A., Tolias G., Avrithis Y., Chum O. Revisiting Oxford and Paris: Large-Scale Image Retrieval Benchmarking. Proceedings of the CVPR, 2018.

[85] Lee S., Seong H., Lee S., Kim E. Correlation Verification for Image Retrieval. Proceedings of the IEEE/CVF Conference on Computer Vision and Pattern Recognition (CVPR).

[86] Yang M., He D., Fan M., Shi B., Xue X., Li F., Ding E., Huang J. DOLG: Single-Stage Image Retrieval with Deep Orthogonal Fusion of Local and Global Features. Proceedings of the IEEE/CVF International Conference on Computer Vision (ICCV).

[87] Cao B., Araujo A., Sim J., Vedaldi A., Bischof H., Brox T., Frahm J.M. (2020). Unifying Deep Local and Global Features for Image Search. Proceedings of the Computer Vision—ECCV 2020.

